# Aramid Nanofiber/MXene-Reinforced Polyelectrolyte Hydrogels for Absorption-Dominated Electromagnetic Interference Shielding and Wearable Sensing

**DOI:** 10.1007/s40820-025-01791-4

**Published:** 2025-05-22

**Authors:** Jinglun Guo, Tianyi Zhang, Xiaoyu Hao, Shuaijie Liu, Yuxin Zou, Jinjin Li, Wei Wu, Liming Chen, Xuqing Liu

**Affiliations:** 1 Center of Advanced Lubrication and Seal Materials, State Key Laboratory of Solidification Processing, Northwestern Polytechnical University, Xi’an, 710072 People’s Republic of China; 2 National Key Laboratory of Scattering and Radiation, Beijing Institute of Environmental Features, Beijing, 100854 People’s Republic of China; 3 Department of Physics and Astronomy, The University of Manchester, Oxford Road, Manchester, M13 9PL UK

**Keywords:** Electromagnetic interference shielding, Intermediate water, Polyelectrolyte hydrogel, Hydrogen bonding, Strain sensor

## Abstract

Conductive hydrogels have garnered widespread attention as a versatile class of flexible electronics. Despite considerable advancements, current methodologies struggle to reconcile the fundamental trade-off between high conductivity and effective absorption-dominated electromagnetic interference (EMI) shielding, as dictated by classical impedance matching theory. This study addresses these limitations by introducing a novel synthesis of aramid nanofiber/MXene-reinforced polyelectrolyte hydrogels. Leveraging the unique properties of polyelectrolytes, this innovative approach enhances ionic conductivity and exploits the hydration effect of hydrophilic polar groups to induce the formation of intermediate water. This critical innovation facilitates polarization relaxation and rearrangement in response to electromagnetic fields, thereby significantly enhancing the EMI shielding effectiveness of hydrogels. The electromagnetic wave attenuation capacity of these hydrogels was thoroughly evaluated across both X-band and terahertz band frequencies, with further investigation into the impact of varying water content states—hydrated, dried, and frozen—on their electromagnetic properties. Moreover, the hydrogels exhibited promising capabilities beyond mere EMI shielding; they also served effectively as strain sensors for monitoring human motions, indicating their potential applicability in wearable electronics. This work provides a new approach to designing multifunctional hydrogels, advancing the integration of flexible, multifunctional materials in modern electronics, with potential applications in both EMI shielding and wearable technology.

**Supplementary Information:**

The online version contains supplementary material available at 10.1007/s40820-025-01791-4.

## Highlights

- Aramid nanofiber/MXene-reinforced polyelectrolyte hydrogels were designed to achieve absorption-dominated electromagnetic interference shielding under the premise of relatively high conductivity.
- The multifunctional composite hydrogels exhibited outstanding mechanical performance, exceptional adhesion strength, excellent electromagnetic interference shielding and reliable capability for monitoring human motion signals.

**Supplementary Information:**

The online version contains supplementary material available at 10.1007/s40820-025-01791-4.

## Graphical abstract

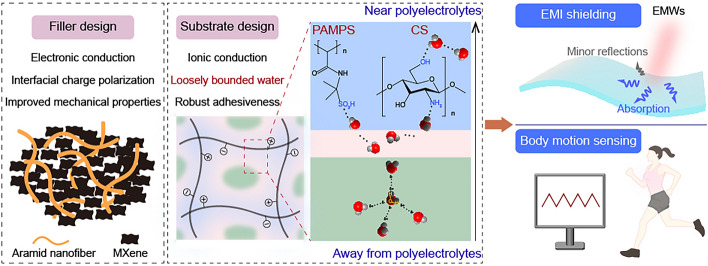

## Introduction

Since entering the new century, humanity has been dedicated to technological innovation in various fields. Among them, electronic technologies and products have lent significant momentum to the advancement of current science and technology [1–5]. Driven by the endless revolution of flexible electronics, recent years have witnessed a spurt progress in the fields of flexible displays [6], flexible batteries [7, 8], intelligent electronic skins [9–11], soft robots [12–14] and, etc. Traditional rigid electronics are being gradually replaced by flexible electronics due to the commensurate softness with human issues, conforming contact with substrate surface and long-term wearability of the latter [15–17]. From the perspective of materials designing, polymer hydrogels can achieve basic flexibility, biocompatibility as well as functionality such as energy storage and sensing in an exquisite manner of engineering [18–21].

The electromagnetic radiation generated by densely distributed electronics will interfere with other surrounding equipment, triggering performance degradation or even operation malfunction [22]. For this reason, it is of a desperate need to develop multifunctional flexible electronics with excellent electromagnetic interference (EMI) shielding performance. As an important component material of flexible electronics, hydrogels have been proved to effectively attenuate electromagnetic waves (EMWs) through multiple reflections and scatterings caused by porous structure, conductive loss of filling networks as well as polarization loss of water and other substances with polar groups [23–26]. The main mechanism of EMI shielding includes reflections on the surfaces of shielding architectures along with inner absorption and multiple reflections. Only a small proportion of EMWs penetrate shielding materials and then continue to propagate. The addition of conductive fillers and the enhancement of the electrical conductivity of hydrogels are crucial means for achieving high-performance hydrogel-based EMI shielding materials. A substantial amount of research work has demonstrated the feasibility of constructing conductive networks with conductive fillers to attenuate EMWs. For instance, Mei et al. [27] enhanced the energy dissipation of EMWs by increasing the content of MXene to simultaneously construct conductive pathways and improve the conductivity of the hydrogel matrix; Li et al. [28] improved the conductivity and EMI shielding performance by optimizing the conductive network structure of poly(3,4-ethylenedioxythiophene):poly(styrene sulfonate) (PEDOT:PSS)/poly(vinyl alcohol) (PVA) dual-network hydrogels using sulfuric acid and MXene; Wang et al. [29] proposed a series of highly conductive hydrogels with metal halides doped PEDOT:PSS as the conductive fillers, designed for applications in thermoelectrics, EMI shielding, Joule heating, etc. Nevertheless, the highly conductive fillers in conductive nanocomposite hydrogels have a large number of free electrons, which do not match the impedance of air, thus leading to significant reflection of EMWs. Notably, strong reflections not only achieve excellent EMI shielding performance, but also account for detrimental secondary electromagnetic pollution [30, 31]. Hence, it is imperative to synthesize absorption-dominated EMI shielding materials. The current strategy is mainly to manipulate the conductivity of hydrogels to optimize impedance matching, which is conducive to the minimization of reflections and transmissions [32, 33]. Unfortunately, the conductivity corresponding to good impedance matching states is often not high enough to meet superior conductivity expectations for many electronics, and reduce the absorption of EMWs at the same time. To this end, it encourages to develop absorption-dominated EMI shielding hydrogels by leveraging composition or structure regulation of absorption under the premise of relatively high conductivity.

Here, an aramid nanofiber (ANF)/Ti_3_C_2_T_x_ MXene-reinforced polyelectrolyte hydrogel has been designed as a versatile wearable electronic, and more importantly as a research model to demonstrate the absorption-dominated EMI shielding mechanism. By leveraging the unique properties of 2-acrylamido-2-methyl-1-propanesulfonic acid (AMPS) and Chitosan (CS), our approach enhanced ionic conductivity and utilized the hydration effect of hydrophilic polar groups to generate intermediate water (IW) with high mobility, which promoted polarization relaxation and rearrangement in response to electromagnetic fields. This innovation rendered it possible to achieve absorption-dominated EMI shielding under the premise of high conductivity. EMI shielding evaluations were conducted on the composite hydrogels across both X-band and terahertz band (THz-band) frequencies. The impact of varying water content states—hydrated, dried, and frozen—on the properties was further investigated. In addition to EMI shielding performance, this hydrogel exhibited outstanding fracture strength and elongation, excellent adhesion, reliable capability for monitoring human motion signals, etc. This work offers a new perspective on the programming of multifunctional integrated hydrogels with absorption-dominated EMI shielding.

## Experimental Section

### Materials

Concentrated hydrochloric acid (HCl), dimethyl sulfoxide (DMSO) and ammonium persulfate (APS) were purchased from Sinopharm Chemical Reagent Co., Ltd. Ti_3_AlC_2_ MAX was acquired from Jilin 11 Technology Co., Ltd. N,N'-Methylenebisacrylamide (MBA), CS, acrylamide (AM), AMPS, potassium hydroxide (KOH) and lithium fluoride (LiF) were bought from Shanghai Aladdin Biochemical Technology Co., Ltd. Agar powder and Luria–Bertani (LB) broth were obtained from Beijing Aoboxing Bio-tech Co., Ltd. Pristine aramid fiber was provided by Tayho Advanced Materials Group Co., Ltd. Ultrapure water was prepared by Ulupure water purification machine. All the chemicals were used as received without any further purification.

### Preparation of Ti_3_C_2_T_x_ MXene

LiF was dissolved in ultrapure water. Then, concentrated HCl was mixed with the LiF solution. The subsequent etching solution was obtained after the uniform blend of the above solution. After that, Ti_3_AlC_2_ MAX (mass ratio: LiF/Ti_3_AlC_2_ = 8/5) was added to the etching solution in small batches. The reaction lasted for 24 h at 40 °C. The resulting dispersion was centrifuged at 3500 r min^−1^ for 5 min per cycle. The washing procedure was repeated for multiple cycles until the pH was ∼6. The upper liquid containing numerous Ti_3_C_2_Tx sheets was collected and then centrifuged at high velocity. The precipitate was dried to obtain Ti_3_C_2_T_x_ MXene.

### Synthesis of ANF Dispersion

KOH was fully dissolved in ultrapure water (150 g mL^−1^). Pristine aramid fiber was entirely immersed in the KOH solution. Then, DMSO (volume ratio: water/DMSO = 1/20) was added to the above solution. The mixture was stirred to completely dissolve the fiber. Ultrapure water was blended with the above dispersion at high stirring speed. The resulting mixture was washed with water until the pH reached neutral. Homogeneous ANF dispersion was obtained after the further stirring.

### Fabrication of A_x_M_y_PC Hydrogels

A_5_M_y_PC (y ≠ 0) hydrogel: ANF dispersion (5 mg mL^−1^) was mixed with Ti_3_C_2_T_x_ MXene. CS, AM and MBA was added to the above mixture. Subsequently, AMPS was blended with the uniformly stirred dispersion. After that, APS was introduced into the mixture. The addition of initiator was followed by the rapid and vigorous stirring. Lastly, the precursor was poured into the glass mold with a spacer and then heated at 60 °C for reaction. A_x_M_y_PC hydrogels were obtained after demolding.

A_0_M_1.5_PC hydrogel: The preparation procedure of A_0_M_1.5_PC hydrogel was similar to that of A_5_M_y_PC (y ≠ 0) hydrogel, except that ANF dispersion was replaced by ultrapure water.

A_5_M_0_PC hydrogel: The synthesis of A_5_M_0_PC hydrogel was similar to that of A_5_M_y_PC (y ≠ 0) hydrogel. The differences were that the precursor did not contain Ti_3_C_2_T_x_ MXene.

A_0_M_0_PC hydrogel: The fabrication of A_0_M_0_PC hydrogel was similar to that of A_5_M_y_PC (y ≠ 0) hydrogel. The differences were that the precursor did not contain Ti_3_C_2_T_x_ MXene and ANF.

### Characterizations

Micro-morphologies were investigated by FEI Helios G4 CX scanning electron microscopy (SEM). Chemical structures were identified by PHI 5000 Versaprobe III X-ray photoelectron spectrometer (XPS), Bruker Tensor II Fourier transform infrared (FTIR) spectrometer, Horiba LabRAM HR Evolution Raman spectrometer and Rigaku SmartLab SE X-ray diffractometer (XRD). Zeta potentials and hydrodynamic sizes were recorded by Malvern Zetasizer Nano Dynamic Light Scattering (DLS) spectrometer. Rheological tests were performed on Haake Mars60 rheometer. The thermal behaviors were recorded from − 80 °C to 150 °C with a heating rate of 5 °C min^−1^ under N_2_ atmosphere using TA Q2000 differential scanning calorimeter (DSC).

### Mechanical Tests

Mechanical properties were characterized using Lyxian HZ-1004B universal testing machine. The uniaxial tensile tests of different hydrogel samples with a dumbbell shape (50 mm in length, 10 mm in gauge length, 4 mm in width and 2 mm in thickness) were performed at a speed of 50 mm min^−1^. The uniaxial compressive tests were conducted on cylindrical samples (16 mm in diameter, 12 mm in height) at a speed of 10 mm min^−1^. For the adhesion tests, the hydrogel samples were prepared into cuboids (20 mm × 20 mm × 2 mm). The adhesion strength was determined by lap shear tests at a speed of 10 mm min^−1^. For the humidity-controlled experiments, an enclosed chamber was constructed around the mechanical testing apparatus, incorporating a humidifier to simulate high-humidity conditions. Hydrogel specimens (HM-A_5_M_1.5_PC) were equilibrated for 15 min under 90% ~ 92% relative humidity before undergoing uniaxial tensile testing. For the elevated-temperature evaluations, samples (HT-A_5_M_1.5_PC) were conditioned in an oven at 40 °C for 2 h prior to mechanical characterization.

### Conductivity Tests

A CHI660E electrochemical workstation was used to record the electrochemical impedance of the hydrogel samples with a frequency range of 10^6^–10^–1^ Hz and a voltage of 100 mV. The ionic conductivity (σ, S·m^−1^) was calculated by the following formula:

1 $$\sigma =L/(R\times S)$$

where L (m) was the thickness, R (Ω) represented the resistance obtained from the electrochemical impedance spectroscopy and S (m^2^) referred to the effective contact area.

### Sensing Performance

Keithley 2450 SourceMeter was employed to acquire resistance signals of hydrogels under different deformations. Two copper wires acting as electrodes were connected to the both ends of hydrogels and the SourceMeter. Gauge factor (GF) was adopted to evaluate the strain sensitivity of the hydrogel sensor. GF was defined as follows:

2 $$GF=(R-{R}_{0})/(\varepsilon \times {R}_{0})$$

where R_0_ (Ω) was the original resistance, R (Ω) was the real-time resistance at a specific strain and ε (%) represented the tensile strain.

### EMI Shielding Performance in the X-Band Range

EMI shielding performance measurements were conducted on Anritsu MS46322B vector network analyzer (VNA) in the X-band range. EMI SE was evaluated through the S parameters.

3 $$R=|{S}_{11}{|}^{2}$$

4 $$T=|{S}_{21}{|}^{2}$$

5 $$A=1-R-T$$

6 $$S{E}_{T}=-10logT$$

7 $$S{E}_{R}=-10log(1-R)$$

8 $$S{E}_{A}=S{E}_{T}-S{E}_{R}$$

where A, R and T was absorption, reflection and transmission coefficients, respectively; SE_T_, SE_R_ and SE_A_ referred to the total, reflection and absorption shielding effectiveness, respectively. The multiple reflection shielding effectiveness (SE_M_) could be ignored when SE_T_ value was larger than 15 dB.

### EMI Shielding Performance in the THz-Band Range

EMI shielding performance measurements were performed on Advantest TAS7400TS terahertz time-domain spectroscopy (THz-TDS) in the THz-band range. The THz pulses were generated using a femtosecond laser with a central wavelength of 1550 nm and a pulse width of 50 fs as the excitation source. The EMI SE values in the THz-band range were calculated based on the following formulas:

9 $$R={E}_{R}^{2}/{E}_{{R}_{0}}^{2}$$

10 $$T={E}_{T}^{2}/{E}_{{T}_{0}}^{2}$$

11 $$A=1-R-T$$

12 $$S{E}_{T}=-10logT$$

13 $$S{E}_{R}=-10log(1-R)$$

14 $$S{E}_{A}=S{E}_{T}-S{E}_{R}$$

where E_T_, E_R_, E_T0_ and E_R0_ was the amplitudes of transmission, reflection, initial transmission and initial reflection of THz pulses, respectively; R, T and A was the absorptivity, transmissivity and reflectivity, respectively. The SE_M_ could be ignored when SE_T_ value was larger than 15 dB.

The THz reflection loss (RL) was calculated according to the following equation:

15 $$RL=-10logR$$

### In Vitro Antibacterial Activity

*Staphylococcus aureus* (*S. aureus*, Gram-positive bacteria) and *Escherichia coli* (*E. coli*, Gram-negative bacteria) were cultured with LB medium in an incubator oscillator at 37 °C overnight to obtain the bacteria suspensions with a concentration of 1 × 10^9^ CFU mL^−1^. The two bacteria suspensions were diluted into 1 × 10^6^ CFU mL^−1^ for subsequent use. The hydrogel samples were cut into round plates with a diameter of 15 mm and then placed into 24-well culture plates with 1.5 mL of diluted bacteria suspension. The culture plates were incubated in an aerobic incubator for 12 h at 37 °C. After that, the samples were gently washed with phosphate-buffered saline (PBS) solution after removing the bacteria suspension. The processed hydrogel samples were placed into centrifuge tubes with 1.5 mL of LB medium. The blender was used to separate bacteria from the hydrogels into centrifuge tubes. 100 μL of separated bacteria suspension was spread onto LB agar plates and then incubated in an aerobic incubator for 24 h at 37 °C. The number of bacteria colonies onto agar plates was recorded using a digital camera.

## Results and Discussion

### Design Principle and Structural Characterizations

**Fig. 1:**
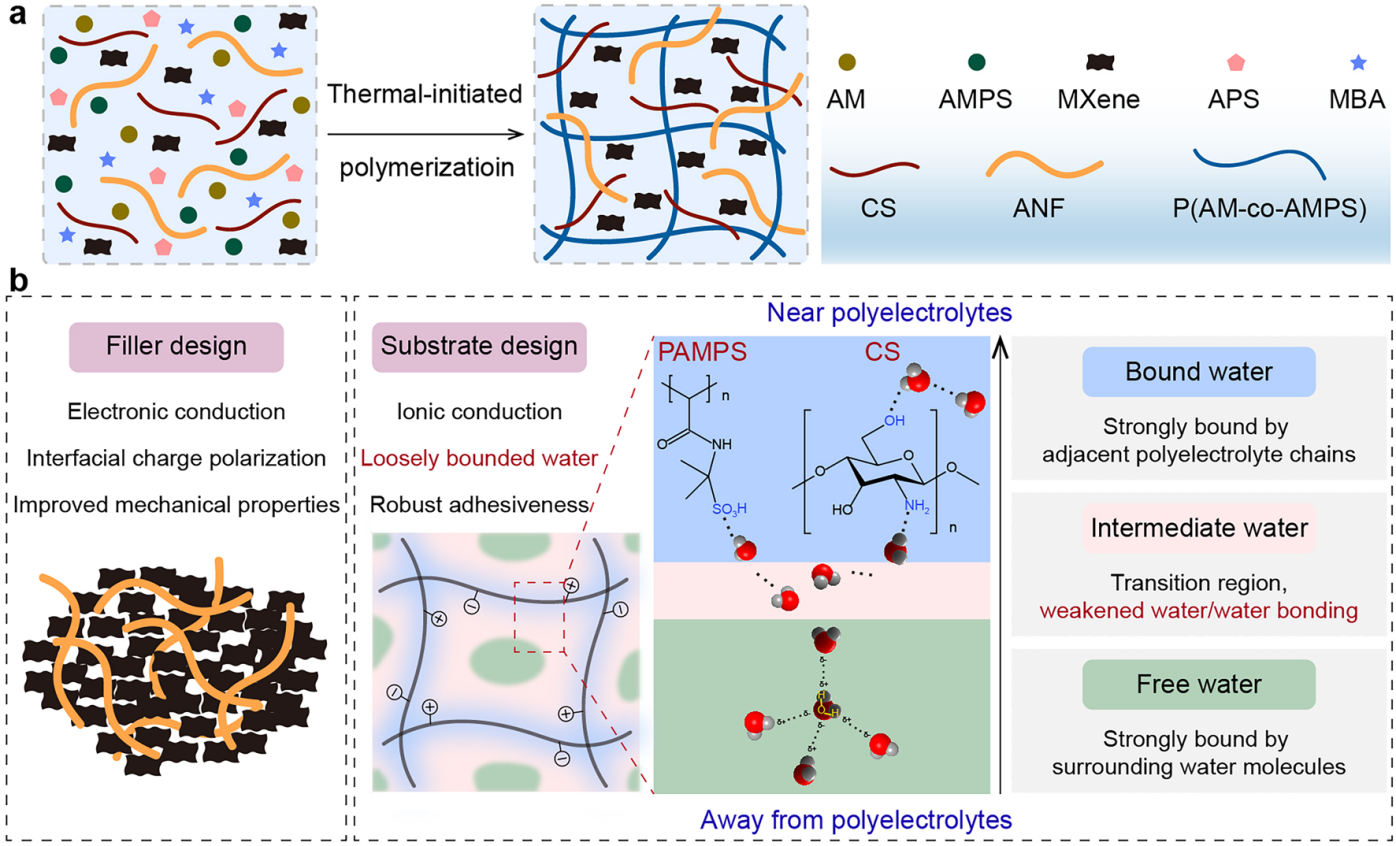
**a** Fabrication process and **b** materials design of ANF/MXene-reinforced polyelectrolyte hydrogel

To fabricate polyelectrolyte hydrogels with favorable mechanical properties, a synthetic protocol was designed based on ANF/Ti_3_C_2_T_x_ MXene-reinforced poly(AM-co-AMPS)/CS (Fig. 1a). The composite hydrogel was denoted as A_x_M_y_PC, where A was ANF, M represented MXene, P referred to the copolymer, C was chitosan. The semi-interpenetrating polymer network poly(AM-co-AMPS)/CS acted as the hydrogel matrix. As depicted in Fig. 1b, the fabrication concept of A_x_M_y_PC hydrogel was designed from the perspective of fillers and polymer matrix. For the filler design, ANF and MXene were incorporated to primarily strengthen the mechanical properties and enhance conductive loss, respectively. Furthermore, the conductivity mismatch between insulated ANF and highly conductive Ti_3_C_2_T_x_ MXene could enhance interfacial charge polarization. With regard to the substrate design, the hydrophilic polar groups of the polyelectrolyte chains, such as -SO_3_H, -NH_2_, and -OH, could help capture the surrounding water molecules through the hydration effect to form bound water (BW) [34, 35]. Water that was far away from the polyelectrolyte polymer chains exhibited almost identical properties to bulk water, hence it was called free water (FW). One FW molecule formed four hydrogen bondings with its adjacent water molecules. The strong interactions between the polyelectrolyte chains and water molecules disturbed the original hydrogen bonding network of FW, thereby resulting in weakly bonded IW molecules located between BW and FW [34, 36]. Numerous studies have proven that IW is a kind of activated water and single IW molecule has more free mobility [34, 37–39]. Activated IW could remarkably weaken the binding effect of the adjacent water on it, conducive to the polarization relaxation under EMWs and rotating arrangement in response to the external electric field. Moreover, the abundant charged ionic groups in the polyelectrolyte chains could introduce ionic conduction, so as to reduce the adverse effect of insulating ANF on the conductivity of the hydrogel. Another important benefit of polyelectrolytes was that sulfonic groups, amino groups and hydroxyl groups endowed hydrogels with a variety of non-covalent interactions with various substrates, including electrostatic attraction, dipole–dipole interactions and hydrogen bondings, to prepare wearable sensors that could be directly adhered to human skin [40, 41]. To sum up, the hydrogel design strategy proposed in this work provides an important alternative for the preparation of multi-functional EMI shielding and wearable sensing materials.

**Fig. 2:**
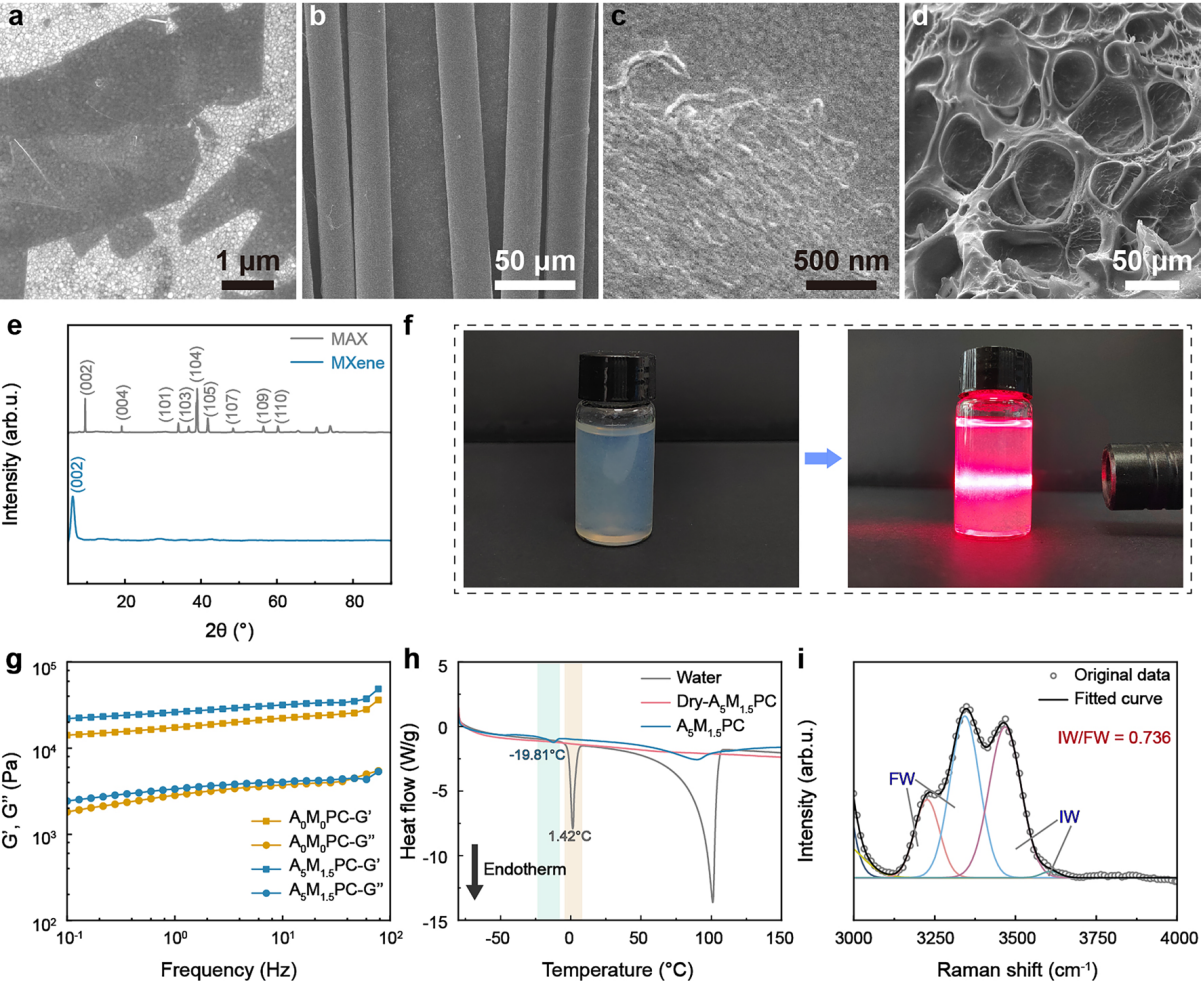
SEM images of **a** Ti_3_C_2_T_x_ MXene, **b** pristine PPTA fiber, **c** ANF and **d** A_5_M_1.5_PC hydrogel. **e** XRD spectra of Ti_3_AlC_2_ MAX and Ti_3_C_2_T_x_ MXene. **f** Dingdall effect of ANF dispersion. **g** Rheological behaviors of A_0_M_0_PC and A_5_M_1.5_PC hydrogel. **h** DSC curves of water, Dry-A_5_M_1.5_PC and A_5_M_1.5_PC hydrogel. **i** Raman spectrum of A_5_M_1.5_PC hydrogel

A series of characterizations were employed to fully study the fundamental characteristics of the polyelectrolyte hydrogel. Ti_3_C_2_T_x_ MXene was synthesized after the removal of Al atoms from Ti_3_AlC_2_ MAX through the classic etching solution consisting of concentrated HCl and LiF (Fig. S1). The as-prepared MXene appeared lamellar structures with varying shapes and sizes (Fig. 2a). The results of XRD (Fig. 2e), XPS (Fig. S2) and Raman (Fig. S3) tests further proved the successful preparation of Ti_3_C_2_T_x_ MXene. The conductive network stacked by the two-dimensional flaky MXene was prone to change with the deformation of hydrogel, achieving the adjustable resistance. ANF dispersion was obtained through the proton donor-assisted deprotonation of poly(*p*-phenylene terephthalamide) (PPTA) fibers (Figs. 2b, f and S4). Hydrophobic interaction triggered the aggregation of ANF in the poor solvent of water when DMSO was completely replaced with water. High concentrations of ANF could form a dense membrane after water evaporation (Fig. 2c). The advantages of stiff ANF as a reinforcing phase in hydrogel matrix have been demonstrated in previous research [42–44]. As shown in Fig. S10a, the pores of A_0_M_0_PC collapsed since no filling was incorporated. MXene strengthened the support to a certain extent (Fig. S10b). The cross section of the hole in ANF-reinforced hydrogel appeared hairy, which was the fiber penetrating the pore wall (Fig. S10c). Dense fiber distribution and the phase separation between ANF and the hydrogel matrix owing to the discrepancies in water affinity was beneficial for boosting the mechanical strength of the composite hydrogel. The polyelectrolyte hydrogel reinforced by ANF and MXene had a more regular pore structure (Fig. 2d). It could be explained by the enhanced support provided by the fillings. Further, the effect of ANF and MXene on the hydrogel matrix was investigated by the rheological tests. Both the storage modulus G′ and loss modulus G″ were enhanced with the addition of the fillings (Fig. 2g). The result revealed that ANF and MXene made a more robust hydrogel structure, which was consistent with the above micromorphology characterizations.

DSC tests were conducted to confirm the distinguishment between the activated water in the composite hydrogel and the bulk water (Fig. 2h). The ice crystals of the bulk water absorbed a lot of heat and melted at around 0 °C. A broad absorption peak located at −19.81 °C in the A_5_M_1.5_PC hydrogel was attributed to the merger of the IW and FW peaks under the high concentration of water. Notably, the strong interaction induced by the directional hydrogen bondings between the polar groups in the hydrogel chains and the water molecules constrained the movement of the water molecules during the cooling process. Consequently, DSC curves failed to detect BW. Moreover, the composite hydrogel with abundant IW would also reduce boiling point. Raman spectra were employed to further qualitatively analyze IW and FW (Fig. 2i). The Raman spectrum of A_5_M_1.5_PC hydrogel in the region of O–H stretching related to the hydrogen bondings between water molecules was deconvoluted into four sub-peaks based on the bonding intensity. The peaks at approximately 3226 cm^−1^ and 3343 cm^−1^ were assigned to FW molecules with two protons and two electron pairs contributing to the formation of hydrogen bondings, while the peaks at around 3468 cm^−1^ and 3608 cm^−1^ were associated with IW molecules that were weakly or non-hydrogen bonded with their adjacent water molecules [38]. According to the three independent tests, the ratios of IW to FW were 0.736, 0.661, and 0.623, respectively (Figs. 2i and S14). Thus, activating water molecules through the polyelectrolyte is an effective and feasible strategy. It reduces the hindrance for the deformation and displacement of water molecules caused by the polarization relaxation in response to electromagnetic fields.

### Mechanical Characterizations of ANF/MXene-Reinforced Hydrogel

**Fig. 3:**
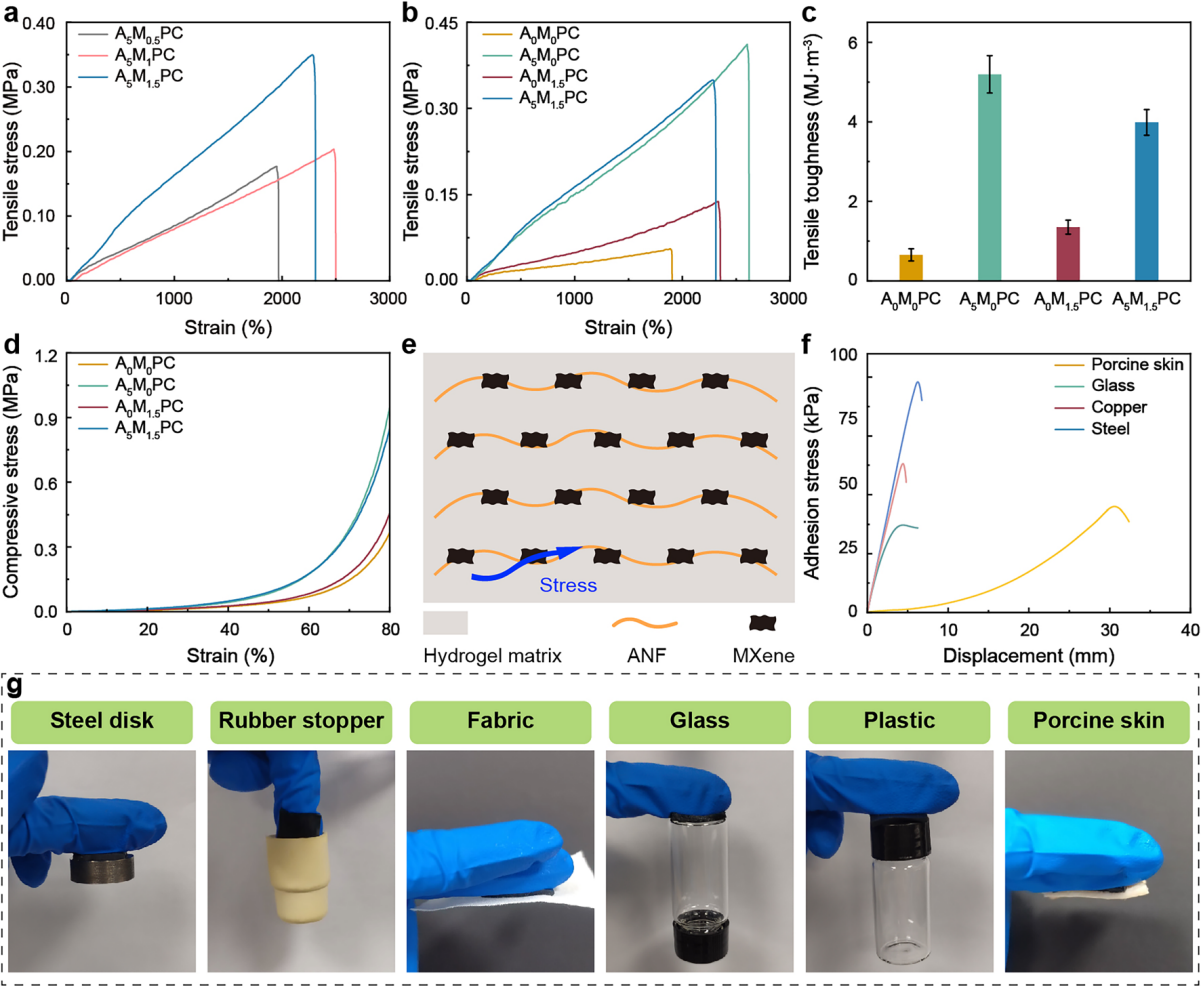
Mechanical characterizations of hydrogels. Uniaxial tensile strain–stress curves of **a** A_5_M_0.5_PC, A_5_M_1_PC and A_5_M_1.5_PC hydrogels as well as** b** A_0_M_0_PC, A_0_M_1.5_PC, A_5_M_0_PC and A_5_M_1.5_PC hydrogels. **c** Tensile toughness and **d** uniaxial compressive strain–stress curves of A_0_M_0_PC, A_5_M_0_PC, A_0_M_1.5_PC and A_5_M_1.5_PC hydrogels. **e** Illustration of stress transference in A_5_M_1.5_PC hydrogel. **f** Lap shear adhesion tests and **g** photos of A_5_M_1.5_PC hydrogel adhered to varied substrates

Mechanical properties are essential criteria to evaluate the resistance of hydrogels to external force. Uniaxial tensile and compressive tests were carried out to examine the contribution of ANF and MXene to the mechanical strength of hydrogel matrix. The tensile strength was gradually boosted with the content of MXene while fixing the volume fraction of ANF (Figs. 3a and S17a). When the mass ratio of MXene to ANF raised from 1:10 to 2:10 and then to 3:10, the increase in tensile strength of the latter was dramatically better than that of the former. This was due to the uneven distribution of crosslinks in the composite network with a small quantity of MXene, accounting for the premature damage of hydrogels by external forces. Moreover, the higher addition of MXene favored the improvement of elongation at break (Fig. S17c). The key factor presumably lied in the fortified crack propagation resistance caused by the two-dimensional lamellar structure of MXene. The loading of MXene did not be further increased considering that the excessive MXene would destroy the continuity of ANF aggregates and augment the reflection loss of EMWs. Single filler-reinforced hydrogels were synthesized to determine the filler that played a dominant role in the improvement of mechanical properties. Compared with A_0_M_0_PC hydrogel, A_5_M_0_PC and A_0_M_1.5_PC hydrogel not only enhanced the tensile strength but the elongation at break (Figs. 3b and S17b, d). Consequently, the tensile toughness was substantially optimized (Fig. 3c). With regard to the uniaxial compressive tests, the contribution of ANF to the improvement of mechanical strength was greater than that of MXene, which was consistent with the uniaxial tensile tests (Fig. 3d). Based on the above analyses, ANF showed more remarkable amelioration effect on the mechanical properties than MXene. A moderate amount of MXene facilitated the homogeneous distribution of composite network. In view of the discrepancies of hydrophilicity and hydrophobicity, the interior of A_5_M_1.5_PC hydrogel was divided into water-rich matrix and hydrophobic ANF-rich phase. Hydrophilic MXene formed firm contacts with ANF and hydrogel matrix through hydrogen bonding, hydrogen bonding and electrostatic interaction, respectively. Therefore, the appropriate amount of MXene could serve as a bridge to fully transfer the stress from hydrogel matrix to reinforcing ANF phase, resulting in optimized mechanical properties (Fig. 3e).

The robust and reliable adhesion of hydrogel is a crucial factor in achieving its seamless integration with human body, thus guaranteeing the high-fidelity acquisitions of body motions and physiological signals. Lap shear tests were conducted to investigate the adhesion strength of A_5_M_1.5_PC hydrogel to the varied substrates (Figs. 3f and S20). Photos of A_5_M_1.5_PC hydrogel adhered to various substrates are showed in Fig. 3g. A_5_M_1.5_PC hydrogel demonstrated distinguished adhesion properties to porcine skin, glass and metals. Among them, the adhesions to the metals were superior to the others. In comparison with the hard substrates, the detachment displacement of relatively soft porcine skin was the largest, and the adhesion strength reached approximately 45 kPa. The vigorous adhesion of A_5_M_1.5_PC hydrogel mainly derived from the numerous non-covalent interactions between the groups of the hydrogel and substrate surfaces. The sulfonate groups in AMPS provided electrostatic adhesion, and formed coordination bondings with metal surfaces. The hydroxyl and amino groups of the polymer chains coupled with the silicon oxide, hydroxyl, carboxyl and sulfhydryl groups through abundant hydrogen bondings, thus endowing A_5_M_1.5_PC hydrogel with powerful adhesion.

### EMI Shielding Performance in the X-Band and THz-Band Range

**Fig. 4:**
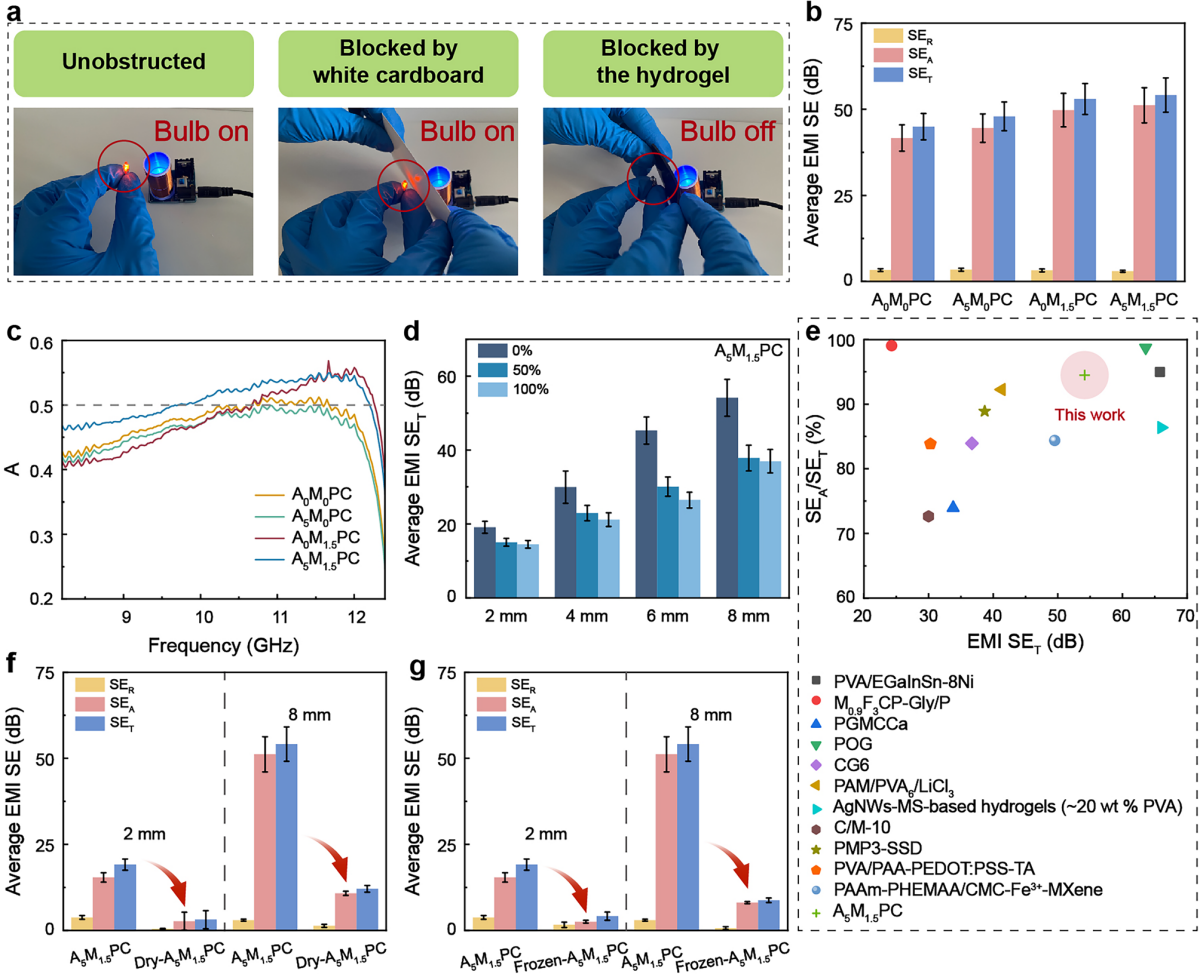
**a** Photos demonstrating the EMI shielding performance of A_5_M_1.5_PC hydrogel through a Tesla coil. **b** Average EMI SE_R_, SE_A_ and SE_T_ of A_x_M_y_PC hydrogels with 8 mm thickness. **c** A values verse frequency curves of A_x_M_y_PC hydrogels with 4 mm thickness. **d** Average EMI SE_T_ of A_5_M_1.5_PC hydrogel with different thicknesses after elongation. **e** Comparison of the ratio of EMI SE_A_ to SE_T_ between this work and other references. Comparison of average EMI SE_R_, SE_A_ and SE_T_ of **f** Dry-A_5_M_1.5_PC and A_5_M_1.5_PC hydrogel as well as **g** Frozen-A_5_M_1.5_PC and A_5_M_1.5_PC hydrogel with the thickness of 2 mm and 8 mm

As an electrically conductive, porous and water-rich material, hydrogels have been proven to be an efficient material to attenuate EMWs through conductive loss, multiple reflections and scatterings by inner walls and strong polarization of water molecules [23, 28, 31]. As shown in Fig. 4a and Movies S1, S2, the EMI shielding performance of the synthesized hydrogel was vividly demonstrated through a Tesla coil and a light bulb. Specifically, a Tesla coil and a light bulb served as an EMWs generator and a receptor, respectively, with materials placed between the two. It could be observed that the white cardboard failed to shield EMWs, while A_5_M_1.5_PC hydrogel effectively shielded EMWs. Further, a series of tests were conducted to systematically and deeply investigate the EMI shielding performance in the X-band range of the composite hydrogel. All the four A_x_M_y_PC hydrogels displayed the significantly enhanced EMI SE_T_ and SE_A_ with the thickness, while the average SE_R_ showed little change (Figs. S22 and S23). Moreover, the percentage of SE_A_ relative to SE_T_ of A_x_M_y_PC hydrogels increased with the thickness, as shown in Fig. S24. Especially, the ratio for A_5_M_1.5_PC hydrogel rose from 80.37% in 2 mm thickness to 94.52% in 8 mm thickness, achieving absorption-dominated EMI shielding for EMWs penetrating into the hydrogel. In addition to thickness, fillings also played an essential role in EMI shielding performance (Figs. 4b and S25). Among the four A_x_M_y_PC hydrogels with different compositions, the A_0_M_0_PC hydrogel without any filling had the lowest EMI SE_T_ and SE_A_ under the same thickness. The A_5_M_0_PC and A_0_M_1.5_PC hydrogel with one type of filling improved the electromagnetic shielding effectiveness (ESE) to a certain extent, wherein MXene showed greater effect than ANF. It was ascribed to the dominant conductive loss caused by the continuous MXene network. The A_5_M_1.5_PC hydrogel with two fillings had the highest ESE. One of the possible factors was the huge disparity in conductivity between MXene and ANF, which was conducive to the multiple inner reflections of EMWs and the interfacial polarization loss. The parameters R, A, and T were calculated to further investigate the underlying electromagnetic shielding mechanism (Figs. 4c and S26). For the A_0_M_0_PC hydrogel, A fluctuated around 0.5 within the 10.75–11.60 GHz range. However, ANF-incorporated formulations exhibited consistently sub-0.5 A values across the X-band. In contrast, MXene addition significantly broadened the effective absorption bandwidth to 10.70–12.25 GHz. Notably, dual-filler hydrogels demonstrated absorption-dominated performance over 9.80–12.20 GHz—covering 57.14% of the X-band spectrum—with an average A exceeding 0.5 throughout the entire X-band. The experimental findings conclusively demonstrated that trace additions of the fillers with highly divergent dielectric properties (ANF: ~ 0.35 wt%, MXene: ~ 0.10 wt%) enabled precise regulation of electromagnetic wave dissipation mechanisms, validating their functions as key engineering parameters in wave energy manipulation. Furthermore, the effect of stretching on EMI shielding performance was evaluated on account of the flexibility of the hydrogel (Figs. 4d and S28, S29). The results illustrated that the ESE including EMI SE_T_, SE_R_ and SE_A_ enhanced with the pristine thickness under the same elongation. Besides, the ESE declined as the stretching rate increased. The root cause of the reduced ESE after stretching lied in the diminished thickness and discontinuous conductive MXene network under large deformation. When the elongation increased from 0% to 50% and then to 100%, the decrease in ESE of the former was greater than that of the latter. The reason was that the thickness change in the central area during the later stage of stretching was weaker than that during the early stage of stretching. In brief, the hydrogels designed in this work attenuated EMWs mainly through absorption. Among them, the SE_A_/SE_T_ of A_5_M_1.5_PC hydrogel was at a relatively advanced level (Fig. 4e).

To elucidate the specific role of water molecules in the EMI shielding performance, relevant tests were carried out on dry and frozen A_5_M_1.5_PC. The ESE slumped when removing water from A_5_M_1.5_PC hydrogel (Figs. 4f and S30). For Dry-A_5_M_1.5_PC, the influence of thickness on the ESE was relatively weak. These results revealed that water was a key factor in the excellent EMI shielding performance of the composite hydrogel. Ultra-low temperature (-80 °C) freezing treatment could make IW and FW crystalize to constrain their polarization relaxation and displacement in response to electromagnetic field. Frozen-A_5_M_1.5_PC had dramatically declined EMI shielding performance, which was consistent with the expected result (Figs. 4g and S31). It meant that the water molecules with weak intermolecular binding force were prone to the attenuation of EMWs. Based on the above analyses, polyelectrolyte chains had a pivotal role in weakening the hydrogen bonding strength between water molecules to optimize EMI shielding performance.

**Fig. 5:**
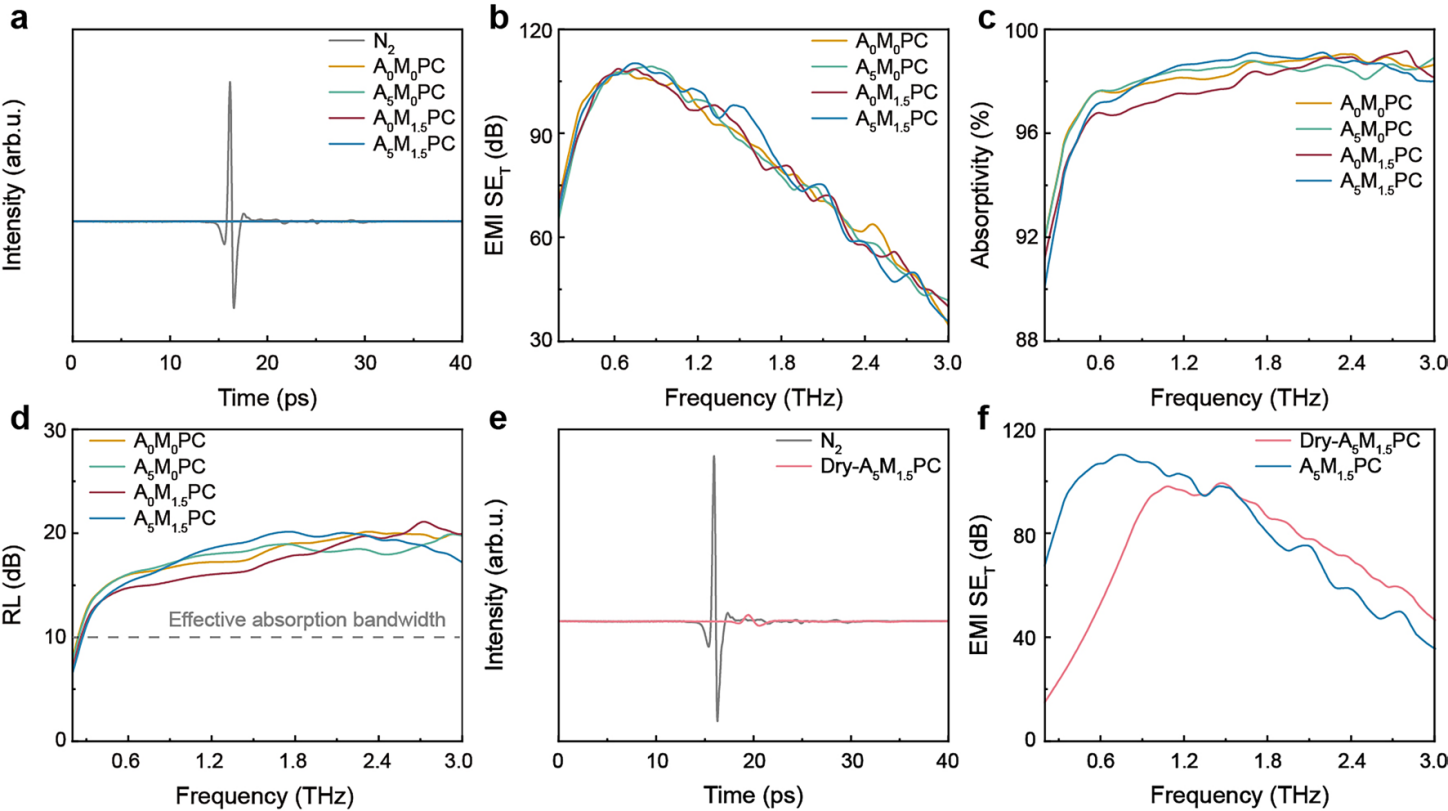
THz EMI shielding performance. **a** THz transmission signals of N_2_ and four various hydrogels. **b** EMI SE_T_, **c** absorptivity and **d** RL of four various hydrogels. **e** THz transmission signals of N_2_ and Dry-A_5_M_1.5_PC hydrogel. **f** EMI SE_T_ of Dry-A_5_M_1.5_PC and A_5_M_1.5_PC hydrogel. The thickness of all the composite hydrogels were 2 mm

Compared with X-band EMWs, THz waves are in a much higher frequency band and occupy an important position in the 6G communication technology. In this respect, there is an urgent need to develop multifunctional materials with both GHz and THz wave absorption characteristics. THz shielding performance of the composite hydrogels and Dry-A_5_M_1.5_PC was explored using THz-TDS system. The THz transmission signals of the four composite hydrogels were overlapping straight lines and far below the incident waves (Fig. 5a). It qualitatively indicated that THz waves could be almost completely shielded in an extremely short period of time. The maximum EMI SE_T_ was approximately 110 dB, and the minimum value exceeded 30 dB in the range of 0.2–3 THz (Fig. 5b). As shown in Fig. S35a, the EMI SE_R_ of all the composite hydrogels was less than 0.5 dB. They achieved high absorptivity of 90%–99% (Fig. 5c). Notably, the fillings had little effect on the EMI shielding performance in the THz-band range, which distinguished from that in the X-band range. It suggested that the hydrogel matrix rather than the fillings played a dominant role in the dissipation of THz waves. Besides, the ESE of THz waves was more significant than that of X-band waves. One possible explanation was that the THz waves with shorter wavelength was much easier to be absorbed and attenuated. More importantly, the vibration and rotation frequency range of most molecules was in the THz-band. The resultant resonance effect greatly boosted the attenuation of EMWs. The wide effective absorption bandwidth (RL > 10 dB) reached 2.7 THz, which also illustrated the efficient THz absorption (Fig. 5d). Further, Dry-A_5_M_1.5_PC was prepared to investigate the influence of water on the THz EMI shielding performance. The test showed that Dry-A_5_M_1.5_PC tremendously consumed the incident THz waves (Fig. 5e). Nevertheless, its transmission signal was still detectable. It was ascribed to the comparatively poor EMI SE_T_ in the low THz range (Fig. 5f). In view of the porous structure of Dry-A_5_M_1.5_PC, it could effectively dissipate THz waves with short wavelength in the relatively higher frequency range even without water. The deep-rooted reason was that the permanent dipoles of water molecules failed to reorient themselves in response to the changes in the high-frequency electromagnetic fields. In other words, water molecules exhibited more significant EMW attenuation ability in the low-frequency range, including X-band and low-frequency part of THz range.

**Fig. 6:**
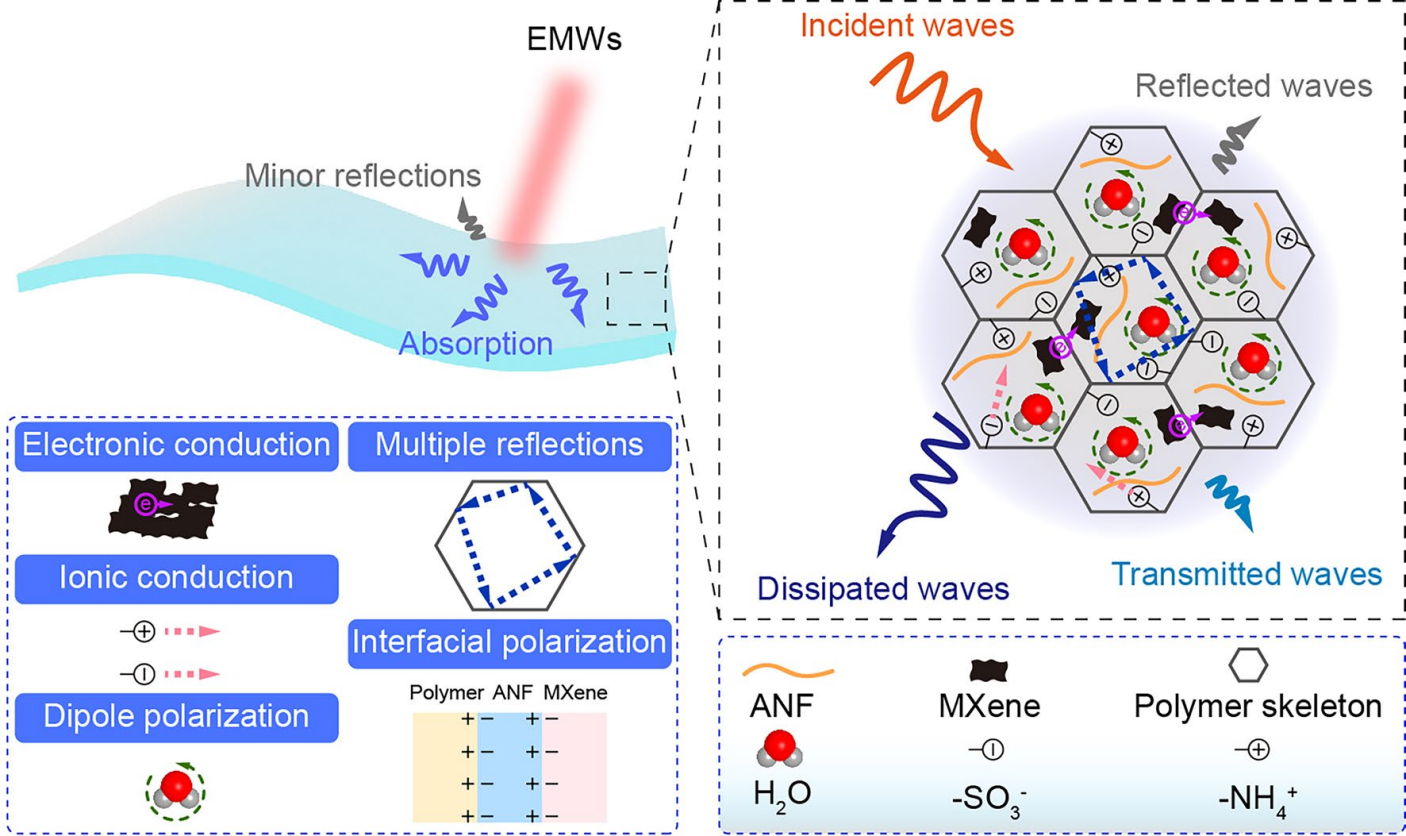
EMI shielding mechanism of A_5_M_1.5_PC hydrogel

To better understand the EMI shielding performance of A_5_M_1.5_PC hydrogel, the corresponding mechanism was summarized and schematically illustrated in Fig. 6 based on the above results. First, the incident EMWs entered the hydrogel surfaces without significant reflection owing to the porous surface structure and moderate conductivity. Then, the transmitted waves propagated inside the hydrogel cells. They underwent multiple reflections and scatterings by conductive MXene network, ANF and polymer skeleton, which contributed to the extension of their propagation paths and the interaction with the cell interfaces before completely penetrating the hydrogel. The conductive MXene network would also expedite the attenuation of EMWs. Besides, the significant conductivity mismatch between MXene, polymer skeleton and ANF effectively promoted the multiple internal reflections and the interface polarization loss. The EMWs that had not been dissipated by the above-mentioned effects accumulated in the water domain. The strong conduction and polarization effect of water molecules greatly consumed EMWs. The hydrophilic MXene with extraordinary thermal conductivity in the water-rich area was conducive to the transfer and dissipation of the heat generated by the eddy current circuits, further advancing the attenuation of EMWs. Importantly, the water molecules in the IW domain weakly bonded with each other. The weak interactions were beneficial to the polarization relaxation of water molecules in contrast with FW. This kind of activated water fortified the dissipation of EMWs through more easily polarization effect. Notably, the vibration and rotation frequencies of most molecules are in THz range. The resulting strong molecular resonance along with the short wavelength characteristic would be the reason for the dramatical attenuation of THz waves in comparison with X-band waves.

### Monitoring of Human Motions

**Fig. 7:**
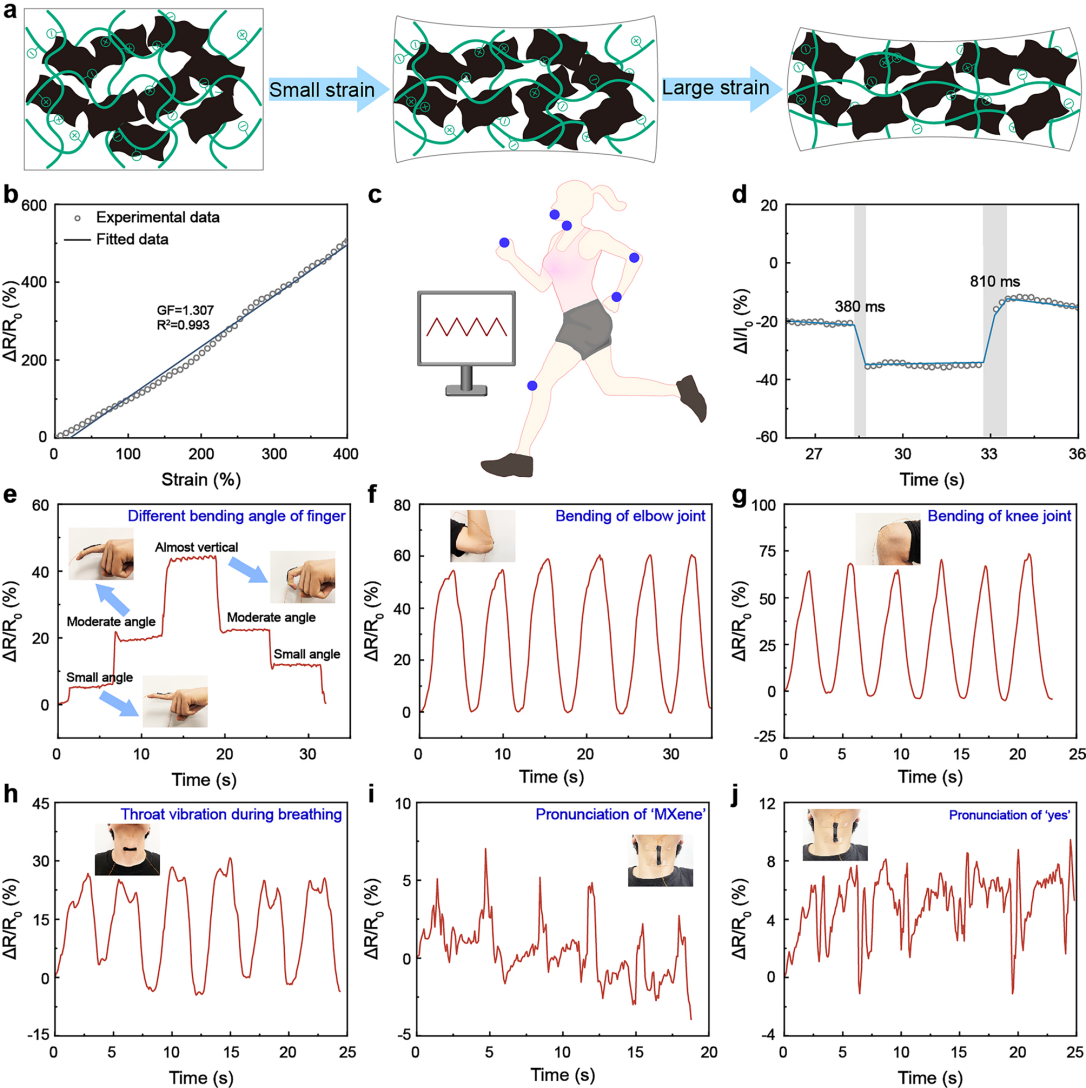
Sensing performance of A_5_M_1.5_PC hydrogel. **a** Mechanism diagram of A_5_M_1.5_PC hydrogels as strain sensors. **b** GF versus consecutive applied tensile strains. **c** Mechanism diagram of A_5_M_1.5_PC hydrogel as a sensor to monitor human motions. **d** Response time and recovery time of A_5_M_1.5_PC hydrogel under electric current stimuli. Human motion monitoring: **e** different bending angle of finger, **f** bending of elbow joint, **g** bending of knee joint, **h** throat vibration during breathing, **i** pronunciation of ‘MXene’, **j** pronunciation of ‘yes’

A_5_M_1.5_PC hydrogel possessed excellent conductivity on account of MXene and the plentiful charged ions in the polymer chains (Fig. S37). Deformation could affect the distribution of MXene nanosheets and caused the movement of charged ions, leading to the change in resistance of A_5_M_1.5_PC hydrogel accordingly (Fig. 7a). Based on the above mechanism, the real-time relative resistance signals of A_5_M_1.5_PC hydrogel under different deformation were recorded to demonstrate its applicability as a strain sensor. The ratio of the change in relative resistance to strain (GF) displayed desirable linear correlation in the tensile strain range of 0–400% (Fig. 7b). It suggested that the electrical signals of the strain sensor could be stably output over a wide strain range. A_5_M_1.5_PC hydrogel was further subjected to current loading and unloading to test its response time and recovery time, which was 380 ms and 810 ms, respectively (Fig. 7d). The changes in relative resistance under varied strain and velocity were measured by a universal testing machine coupled with a digital source meter (Fig. S38). A_5_M_1.5_PC hydrogel showed symmetric signal response in the repeated stretching and releasing process. It verified that A_5_M_1.5_PC sensor had repeatable and reliable workability. Notably, its detection limit of strain signals was as low as 1%. Sticky A_5_M_1.5_PC hydrogel was attached on the different parts of the volunteer’s body to acquire the instant response signals of human motion. The tensile and compressive strains caused by finger bending triggered the change in resistance of the hydrogel. It meant that the relative resistance varied with the bending angle of finger (Fig. 7e). The resistance maintained at a constant level when holding the finger at a certain bending angle. The relative resistance reduced to its initial value as the finger gradually returned to the extended state. It proved that the resistance signals monitored by the A_5_M_1.5_PC hydrogel could fully reflected the finger bending behaviors. The hydrogel was placed at elbow, knee and wrist joint to detect human motion signals with large strains (Figs. 7f, g and S39a). It was found that different body motions brought about different resistance responses, and the sensor presented repeatable sensing responsiveness. The results indicated the robust sensing capability of A_5_M_1.5_PC strain sensor. The hydrogel attached on the throat could precisely detect subtle human motions. The throat produced different vibrations when pronouncing ‘MXene’, ‘yes’ and ‘flexible’ (Figs. 7h-j and S39b). These signals were easily captured by the hydrogel sensor. The pronunciation of varied words corresponded to the distinguishable signal waveforms, indicating the sensitivity of A_5_M_1.5_PC hydrogel to throat vibrations. The electrical signals in the above tests showed a synchronous change with human motions without any lag. Hence, A_5_M_1.5_PC hydrogel was proved to be a potential candidate as a highly sensitive strain sensor for real-time body motion monitoring.

## Conclusions

In this paper, ANF/MXene-reinforced polyelectrolyte composite hydrogel was reasonably designed and fabricated for versatile flexible electronics. The mechanical characterizations showed the positive role of ANF and MXene as the reinforcing phase of the hydrogel matrix. A series of tests verified the formation of IW originated from the hydration effect of the polyelectrolyte chains along with their weakening hydrogen bondings with the adjacent water molecules. Based on the activated water molecules, EMI shielding properties of the composite hydrogel were explored, and the effect of water was further studied after drying and freezing the samples. Most importantly, water molecules exhibited more significant EMWs attenuation ability in the low-frequency range, including X-band and low-frequency part of THz range. The porous structure, conductive network, mismatch in conductivity among components coupled with enhanced polarization and mobility accounted for the distinguished EMI shielding performance. It is noteworthy that the shielding performance in the X-band range was inferior to that in the THz-band, which was owing to the easier attenuation of THz waves with shorter wavelengths. Besides, some tests were carried out to demonstrate its potential as a strain sensor for monitoring human motion signals. This study provides a new perspective for the synthesis of versatile flexible electronics with exceptional EMI shielding performance based on hydrogels.

## Supplementary Information

Below is the link to the electronic supplementary material.

Supplementary file1 (MP4 14007 kb)

Supplementary file2 (MP4 10167 kb)

Supplementary file3 (DOCX 5064 kb)
