# Supplementary material for: Aramid Nanofiber/MXene-Reinforced Polyelectrolyte Hydrogels for Absorption-Dominated Electromagnetic Interference Shielding and Wearable Sensing
Source: Nanomicro Lett. 2025 May 22;17:271. doi: 10.1007/s40820-025-01791-4 (PMC12098229; doi:10.1007/s40820-025-01791-4)
Supplement: Supplementary file 3 — Supplementary file3 (DOCX 5064 kb) [file 40820_2025_1791_MOESM3_ESM.docx]

Supporting Information for

**Aramid Nanofiber/MXene-Reinforced Polyelectrolyte Hydrogels for Absorption-Dominated Electromagnetic Interference Shielding and Wearable Sensing**

Jinglun Guo ^1^, Tianyi Zhang ^1^, Xiaoyu Hao ^1^, Shuaijie Liu ^1^, Yuxin Zou ^1^, Jinjin Li ^2^, Wei Wu ^2^, Liming Chen ^3,^ *, Xuqing Liu ^1,^ *

^1^ Center of Advanced Lubrication and Seal Materials, State Key Laboratory of Solidification Processing, Northwestern Polytechnical University, Xi’an, 710072, P. R. China

^2^ National Key Laboratory of Scattering and Radiation, Beijing Institute of Environmental Features, Beijing, 100854, P. R. China

^3^ Department of Physics and Astronomy, The University of Manchester, Oxford Road, Manchester, M13 9PL, UK

*Corresponding authors. E-mail: xqliu@nwpu.edu.cn (Xuqing Liu); liming.chen@manchester.ac.uk (Liming Chen)

**Supplementary Figures and Tables**


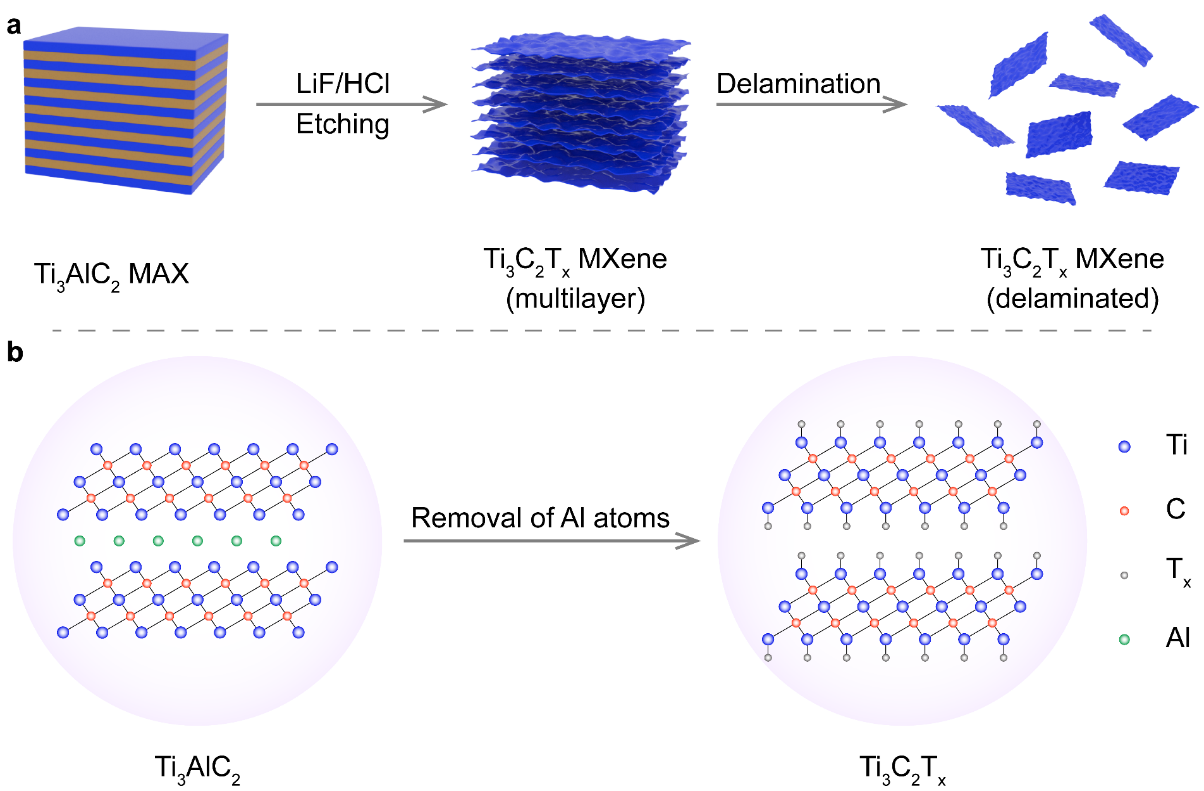


**Fig. S1** Schematic diagram of the fabrication of Ti_3_C_2_T_x_ MXene. **a** The main synthesis procedures of delaminated Ti_3_C_2_T_x_ MXene. **b** The structural change of Ti_3_AlC_2_ MAX before and after the acid treatment


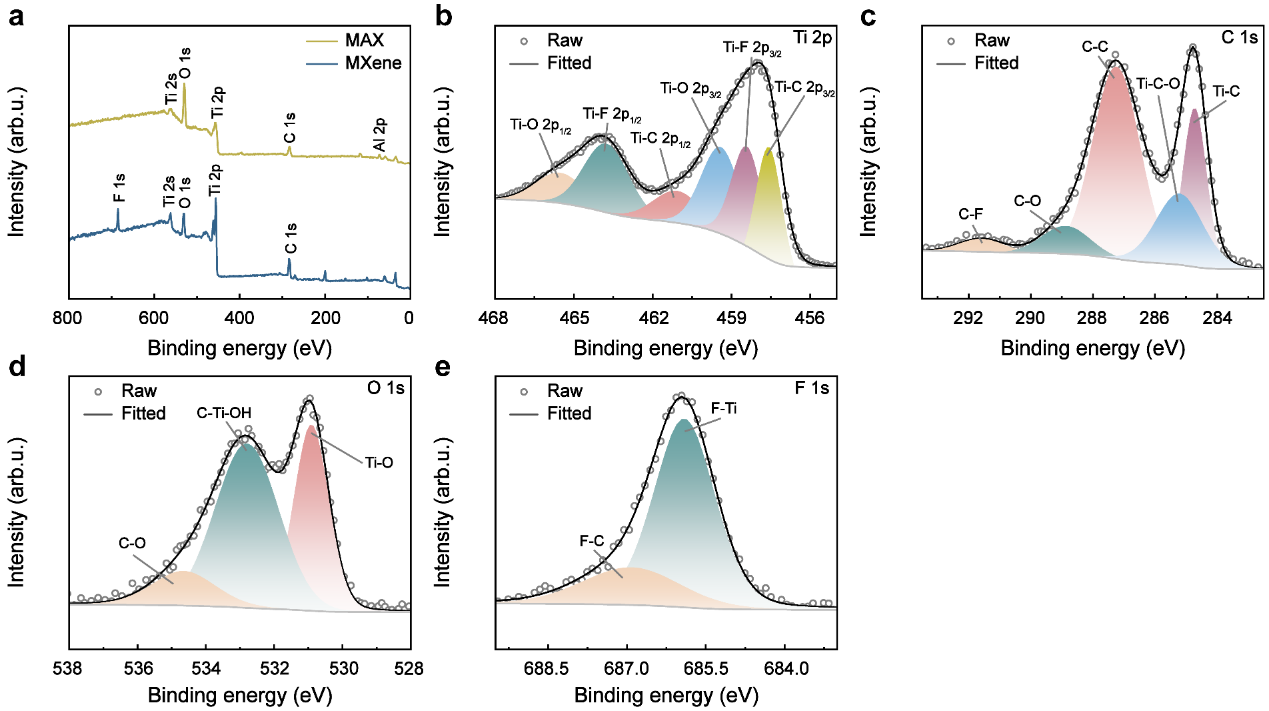


**Fig. S2** **a** XPS survey spectra of Ti_3_AlC_2_ MAX and Ti_3_C_2_T_x_ MXene. The high-resolution XPS spectra of **b** Ti 2p, **c** C 1s, **d** O 1s and **e** F 1s in MXene

As depicted in Fig. S2a, Al element signals faded accompanied by the appearence of F element in the survey spectrum of Ti_3_C_2_T_x_ MXene compared with that of Ti_3_AlC_2_ MAX, indicating that the successful removal of Al atoms from Ti_3_AlC_2_ MAX and the formation of -F groups. The high resoltion spectra of Ti 2p, C 1s, O 1s and F 1s in MXene were shown in Fig. S2b-e.


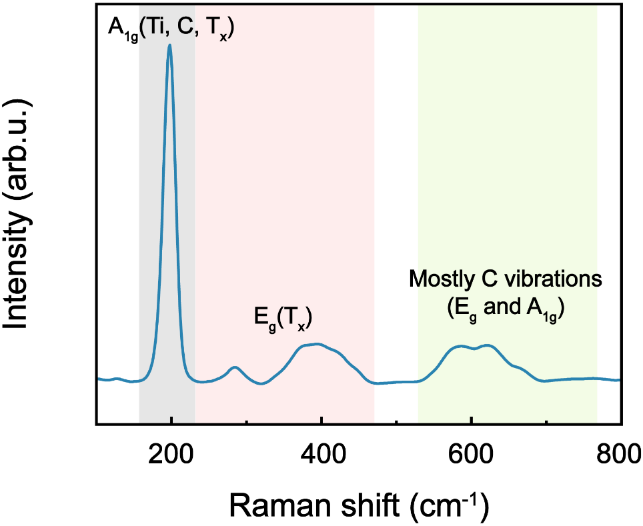


**Fig. S3** Raman spertrum of MXene

The peak at 197.7 cm^-1^ was assigned to the typical out-of-plane (A_1g_) vibrations of Ti atoms in the outer layer along with carbon and surface groups^[1]^. Further, the broad peaks in the region of 230~470 cm^-1^ was designated as in-plane (E_g_) modes of surface groups attached to Ti atoms [S1, S2]. The peak region between 530 cm^-1^ and 765 cm^-1^ was associated mostly with carbon vibrations (both E_g_ and A_1g_) [S1].


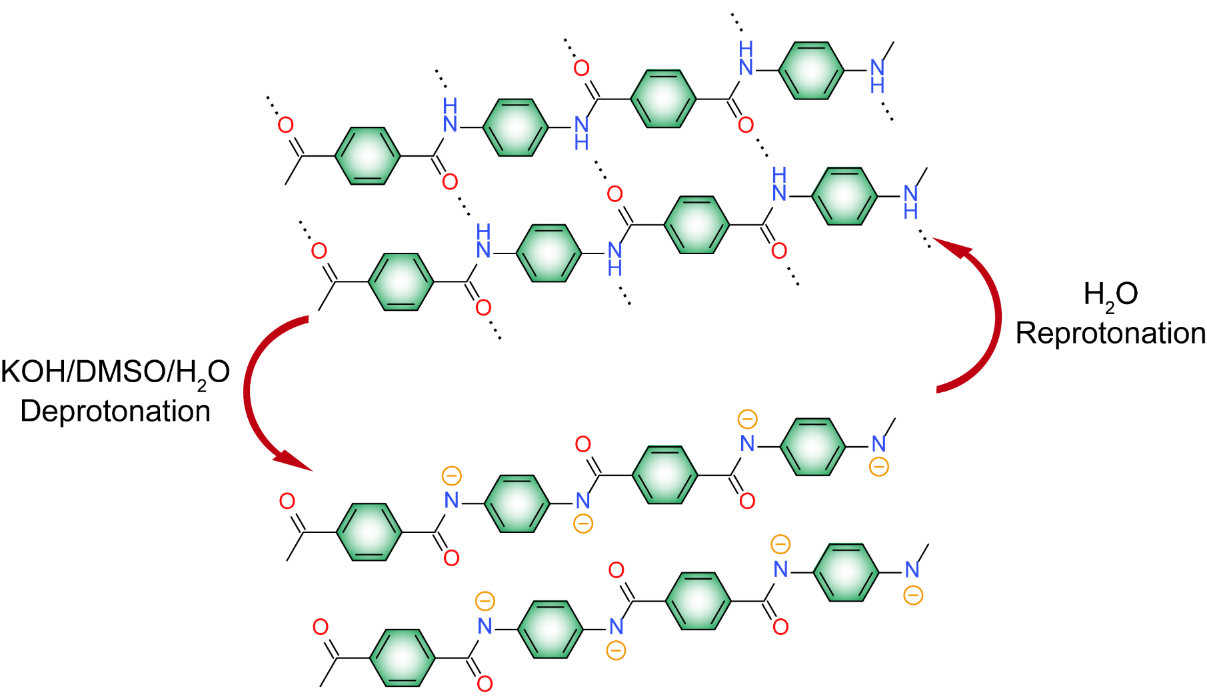


**Fig. S4** Atomic structures of deprotonation and reprotonation in aramid fibers


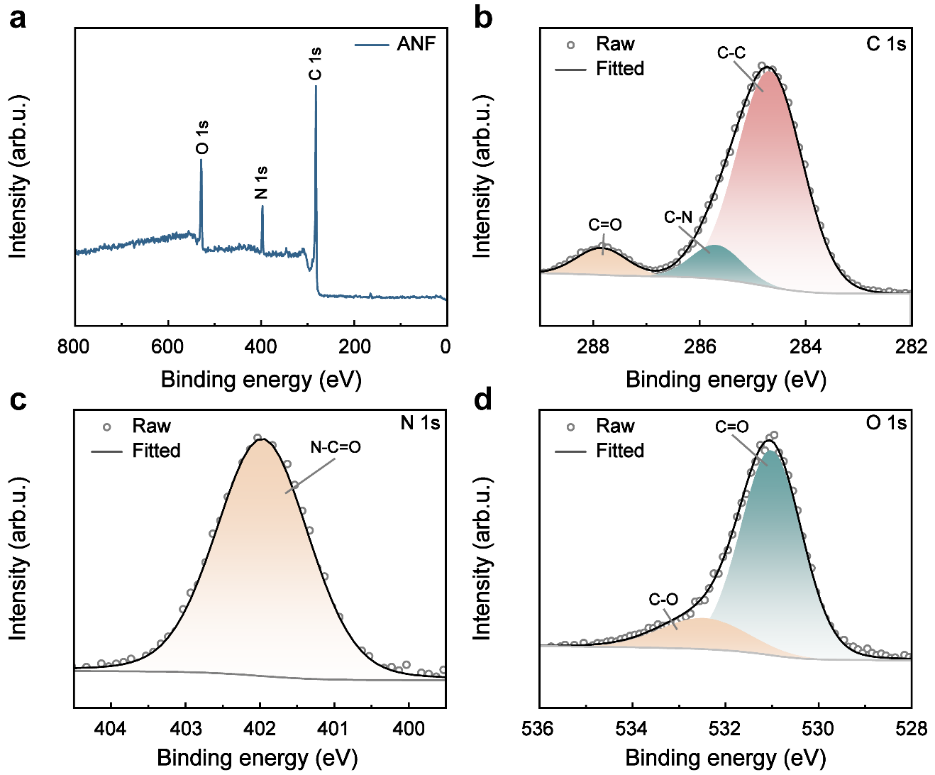


**Fig. S5** **a** XPS survey spectrum of ANF. The high-resolution XPS spectra of **b** C 1s, **c** N 1s and **d** O 1s in ANF

The survey spetrum manifested that ANF was composed of C, N and O elements. The peaks of C 1s at 287.8 eV, N 1s at 402.0 eV and O 1s at 531.0 eV were assigned to -CO-NH- group.


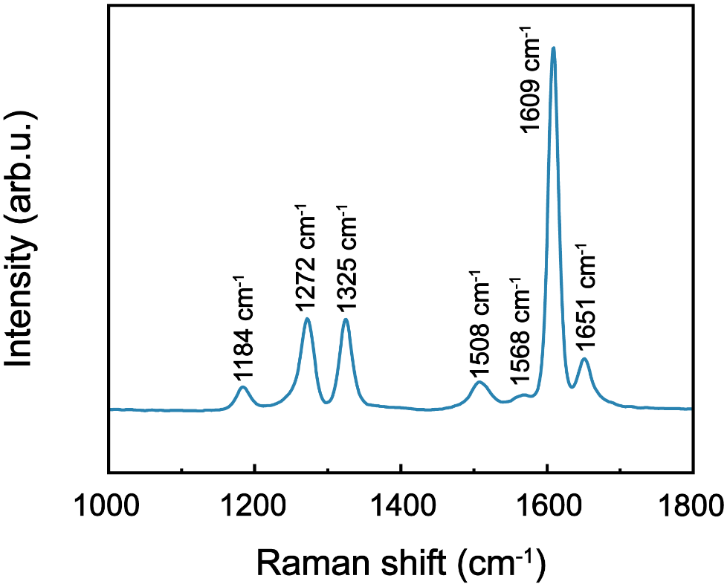


**Fig. S6** Raman spertrum of ANF

The peak at 1568 cm^-1^ was associated with the coupled C-N in-plane deformation and C-N stretching modes [S3]. The absorption peak located at 1609 cm^-1^ was related to the C=C stretching band of the benzene ring of ANF [S4].


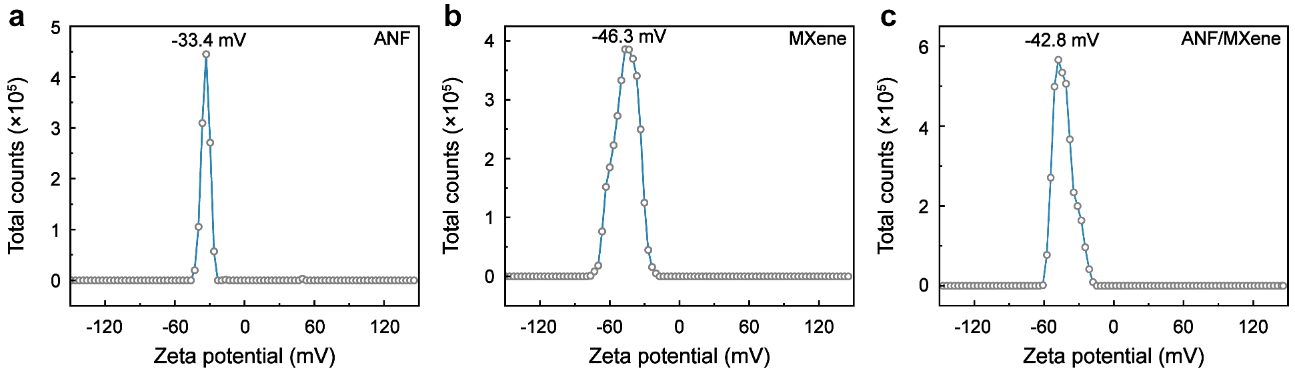


**Fig. S7** Zeta potential of **a** ANF, **b** MXene and **c** ANF/Mxene


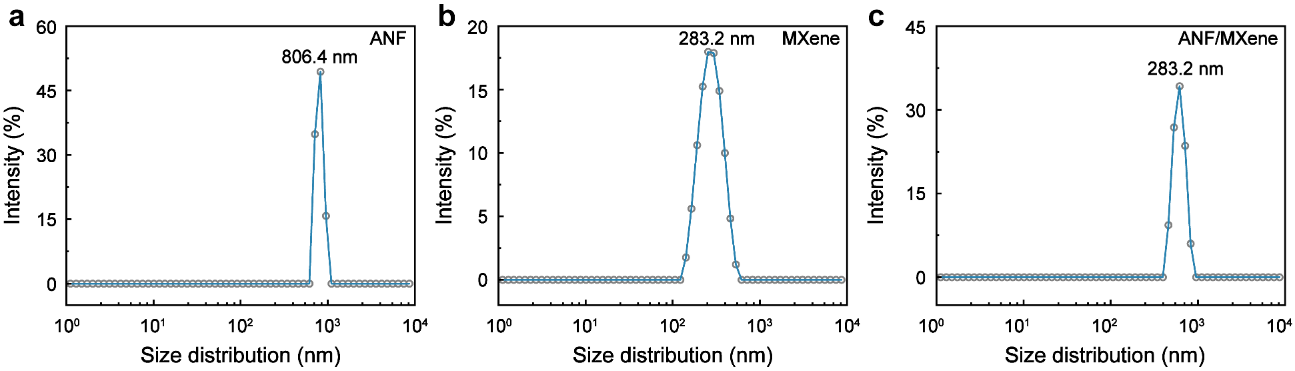


**Fig. S8** Hydrodynamic size of **a** ANF, **b** MXene and **c** ANF/MXene


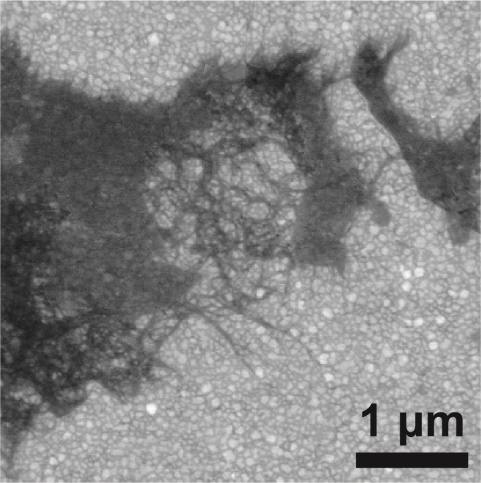


**Fig. S9** SEM image of ANF/Mxene


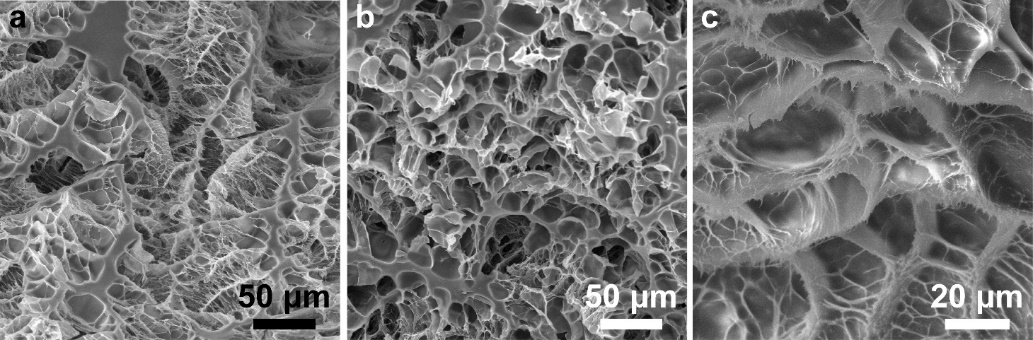


**Fig. S10** SEM images of **a** A_0_M_0_PC, **b** A_0_M_1.5_PC and **c** A_5_M_0_PC


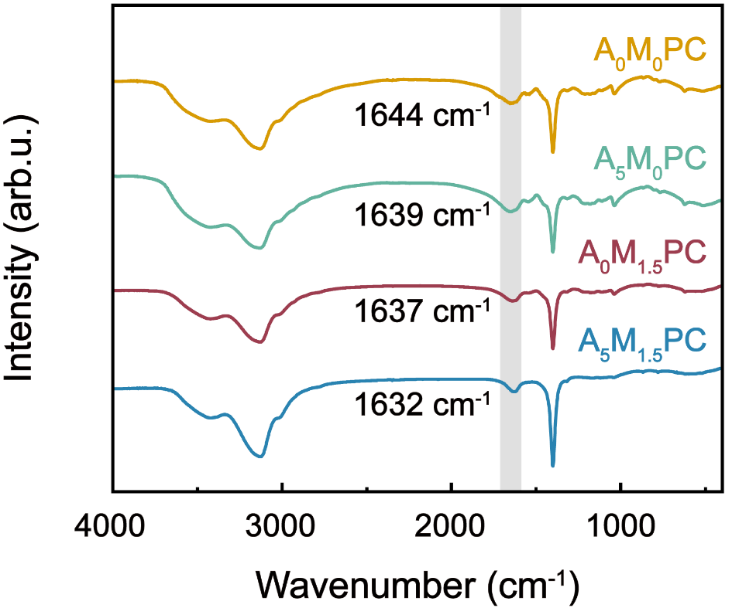


**Fig. S11** FTIR spectra (TR mode) of A_0_M_0_PC, A_5_M_0_PC, A_0_M_1.5_PC and A_5_M_1.5_PC hydrogels

After incorporating ANF and MXene, the peak at 1644 cm^-1^ shifted to 1632 cm^-1^, i.e. red shift. It indicated the formation of hydrogen bondings near amide groups.


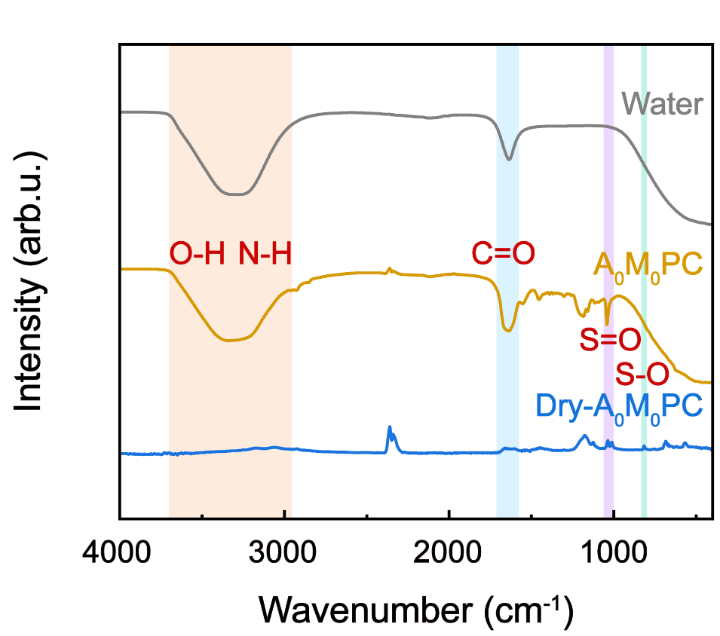


**Fig. S12** FTIR spectra (ATR mode) of Water, A_0_M_0_PC, and Dry-A_0_M_0_PC hydrogels

The spectral profile of pure water was analyzed to characterize hydrogen bonding interactions in free water (FW) molecules, establishing reference peaks for subsequent analytical procedures. A broad and intense O-H stretching vibration peak observed at ~3300 cm^-1^ arose from the formation of an intermolecular hydrogen-bonding network in FW. The A_0_M_0_PC hydrogel exhibited a markedly broadened O-H stretching band with a redshift to ~3250 cm^-1^, superimposed with the N-H vibration (~3300 cm^-1^) originating from chitosan (CS). Water molecules formed multiple hydrogen bondings with polar functional groups (-OH, -NH_2_, -SO_3_H) within the polymer matrix. The underlying interaction mechanisms involved: (i) strong hydrogen bonding between chitosan’s -OH and -NH_2_ groups with water molecules, inducing spectral broadening and shifting; (ii) reinforced hydrogen-bonding networks through N-H···O interactions between acrylamide’s (AM’s) -CONH_2_ and water molecules. The freeze-dried hydrogel (Dry-A_0_M_0_PC) demonstrated significant attenuation in O-H peak intensity due to the removal of FW, partial or complete elimination of IW, and partial extraction of bound water (BW). While the predominant removal of water molecules led to partial dissociation of the hydrogen-bonding network, inherent vibration peaks of the polymer matrix became discernible in the spectral profile. Overall, water molecules formed multiple hydrogen bondings via the O-H groups with the -OH and -NH_2_ in CS, the -CONH_2_ in AM, and the -SO_3_H in AMPS, collectively establishing a dynamic cross-linked network. However, IW served as a transitional water molecule, exhibiting binding strength with the polymer matrix that was intermediate between that of FW and BW. Therefore, it remains challenging to precisely differentiate IW, FW, and BW based on FTIR test results. Nevertheless, the FTIR analyses still provides a vital characterization tool for investigating interactions between water molecules and the polymer matrix.


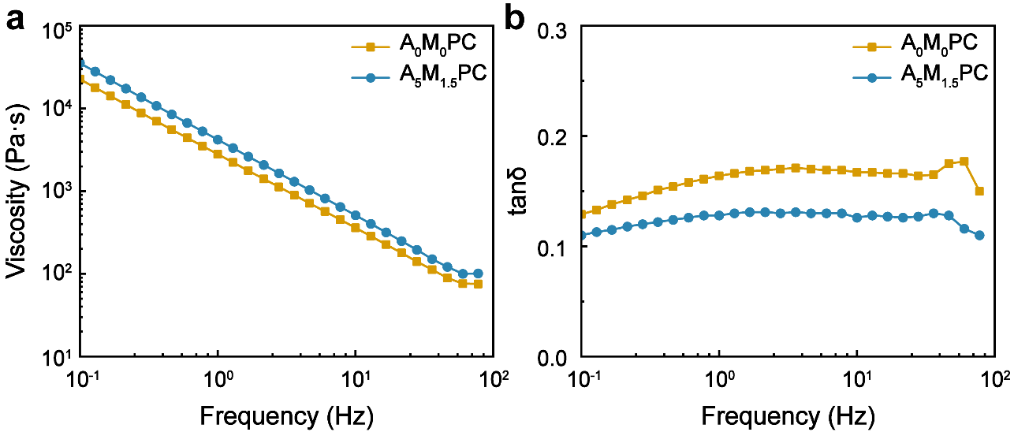


**Fig. S13** Rheological properties of A_0_M_0_PC and A_5_M_1.5_PC hydrogels. **a** The variations of viscosity with frequency. **b** The loss tangent at different frequencies

The viscosity of A_5_M_1.5_PC hydrogel was always higher than that of A_0_M_0_PC hydrogel at all frequencies, indicating that the addition of ANF and MXene could improve the viscosity of the hydrogel matrix. The viscosity of both A_0_M_0_PC and A_5_M_1.5_PC hydrogel was dramatically descended with the frequency. It could be explained by the fracture of the hydrogel network. The loss tangent (tanδ) of both A_0_M_0_PC and A_5_M_1.5_PC hydrogel was below 0.2 at all frequencies, suggesting that the elasticity was dominant in the viscoelastic hydrogels with the strong molecular interaction. Moreover, ANF and MXene further enhanced the elasticity.


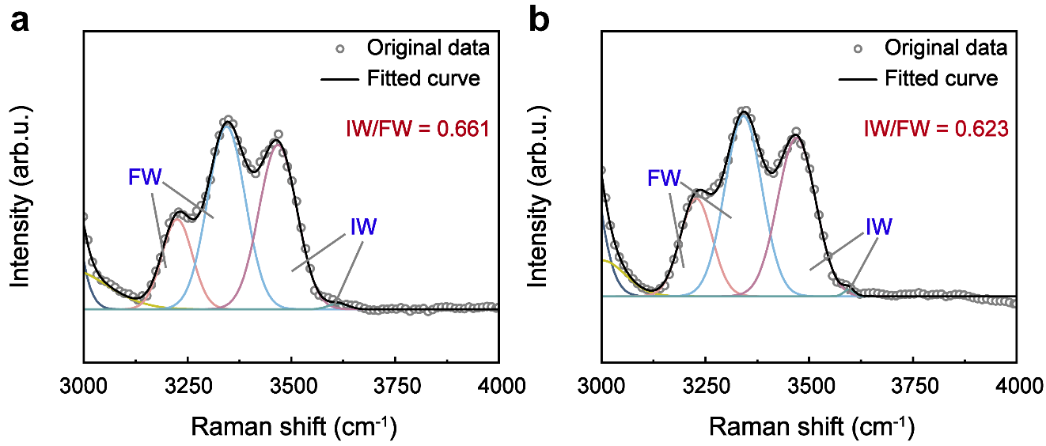


**Fig. S14** Raman spectra of A_5_M_1.5_PC hydrogel in different spots


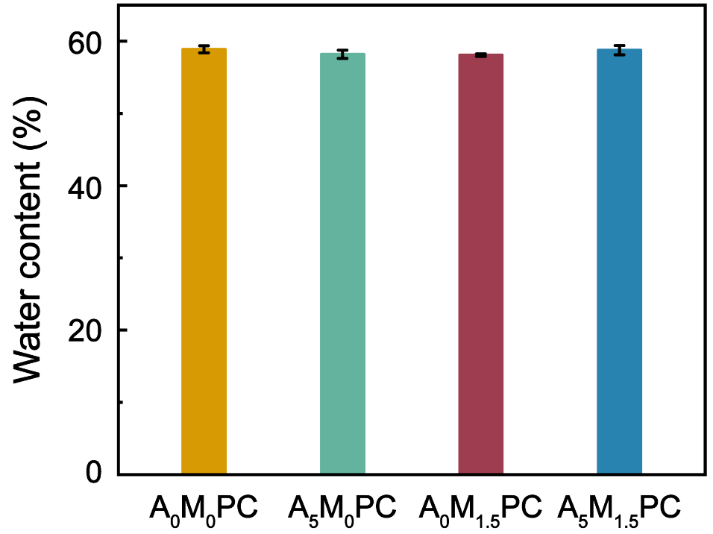


**Fig. S15** Water content of A_0_M_0_PC, A_5_M_0_PC, A_0_M_1.5_PC and A_5_M_1.5_PC hydrogels


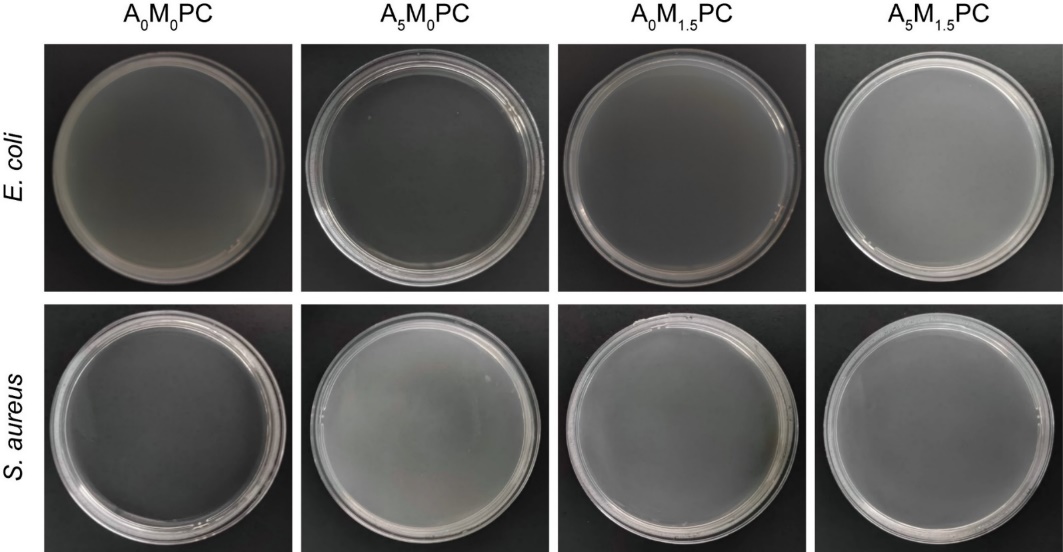


**Fig. S16** Antibacterial activity of A_0_M_0_PC, A_5_M_0_PC, A_0_M_1.5_PC and A_5_M_1.5_PC hydrogels


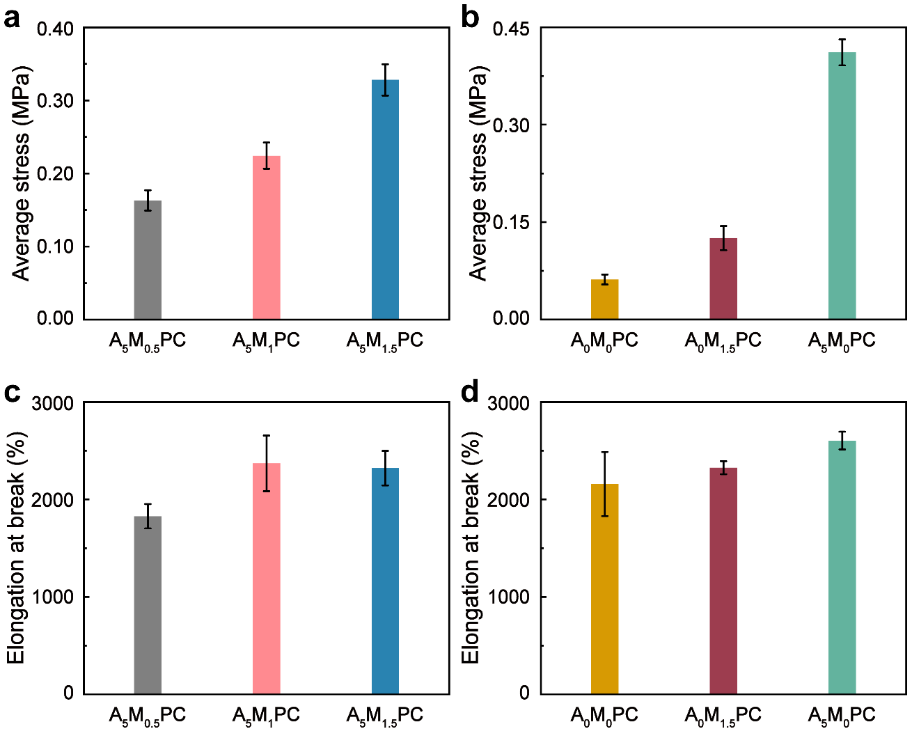


**Fig. S17** Average tensile stress of **a** A_5_M_0.5_PC, A_5_M_1_PC and A_5_M_1.5_PC as well as **b** A_0_M_0_PC, A_0_M_1.5_PC and A_5_M_0_PC hydrogels. Average elongation at break of **c** A_5_M_0.5_PC, A_5_M_1_PC and A_5_M_1.5_PC as well as **d** A_0_M_0_PC, A_0_M_1.5_PC and A_5_M_0_PC hydrogels


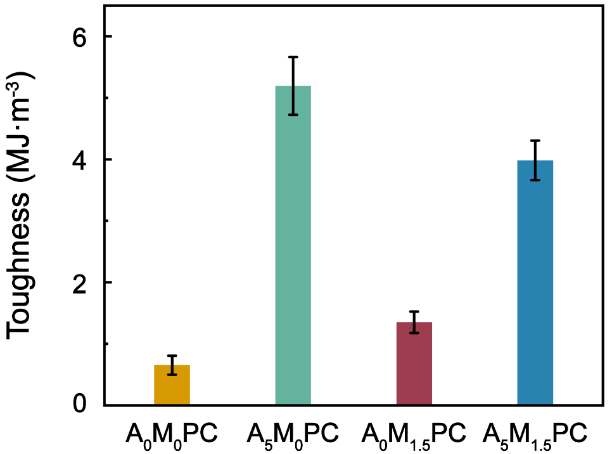


**Fig. S18** Tensile toughness of A_0_M_0_PC, A_5_M_0_PC, A_0_M_1.5_PC and A_5_M_1.5_PC hydrogels


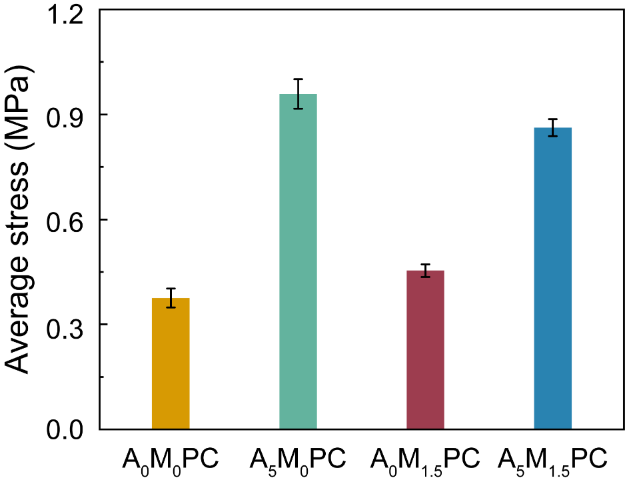


**Fig. S19** Average compressive stress of A_0_M_0_PC, A_5_M_0_PC, A_0_M_1.5_PC and A_5_M_1.5_PC hydrogels


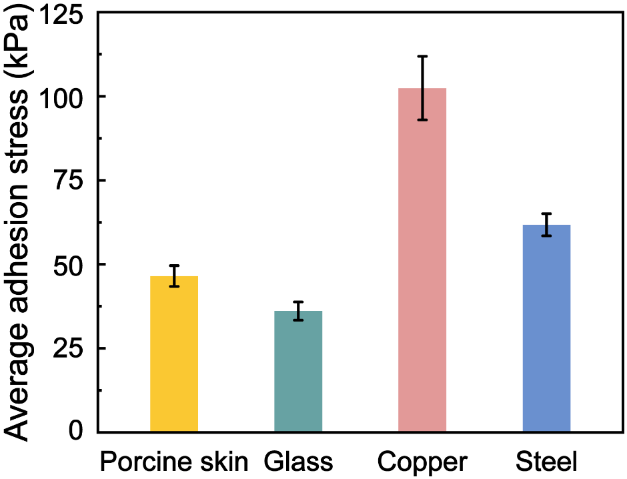


**Fig. S20** Average adhesion stress of A_5_M_1.5_PC hydrogel to different substrates


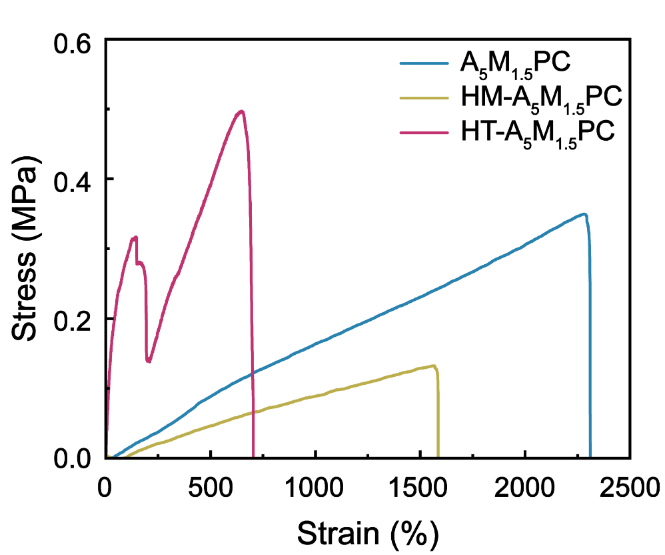


**Fig. S21** Uniaxial tensile strain-stress curves of A_5_M_1.5_PC, HM-A_5_M_1.5_PC and HT-A_5_M_1.5_PC

Under elevated temperatures, accelerated dehydration in hydrogels led to progressive rigidity through flexibility loss. Partial water depletion enhanced tensile strength but reduced fracture elongation. Strain-stress curves demonstrated non-monotonic progression with oscillatory patterns during stretching, attributed to modulus mismatch between rapidly dehydrated surface layers and hydrated internal regions exposed through mechanical extension.

Under high-humidity conditions, hydrogel specimens absorbed ambient moisture, exhibiting slight volumetric expansion and structural relaxation. This phenomenon led to near-zero stress levels during the initial strain phase of the stress-strain curve.

Therefore, based on the mechanical performance testing under extreme temperature and humidity conditions outlined above, the synthesized hydrogel materials in this study are recommended for application in environments with moderate thermal and hygroscopic parameters.


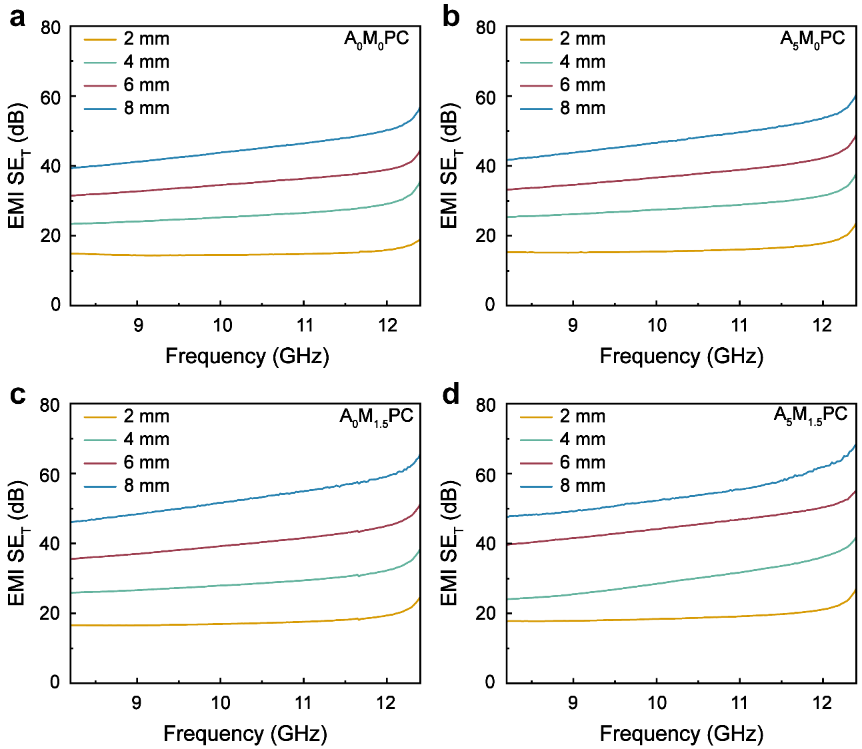


**Fig. S22** EMI SE_T_ curves of **a** A_0_M_0_PC, **b** A_5_M_0_PC, **c** A_0_M_1.5_PC and **d** A_5_M_1.5_PC hydrogels with different thicknesses in the X-band range


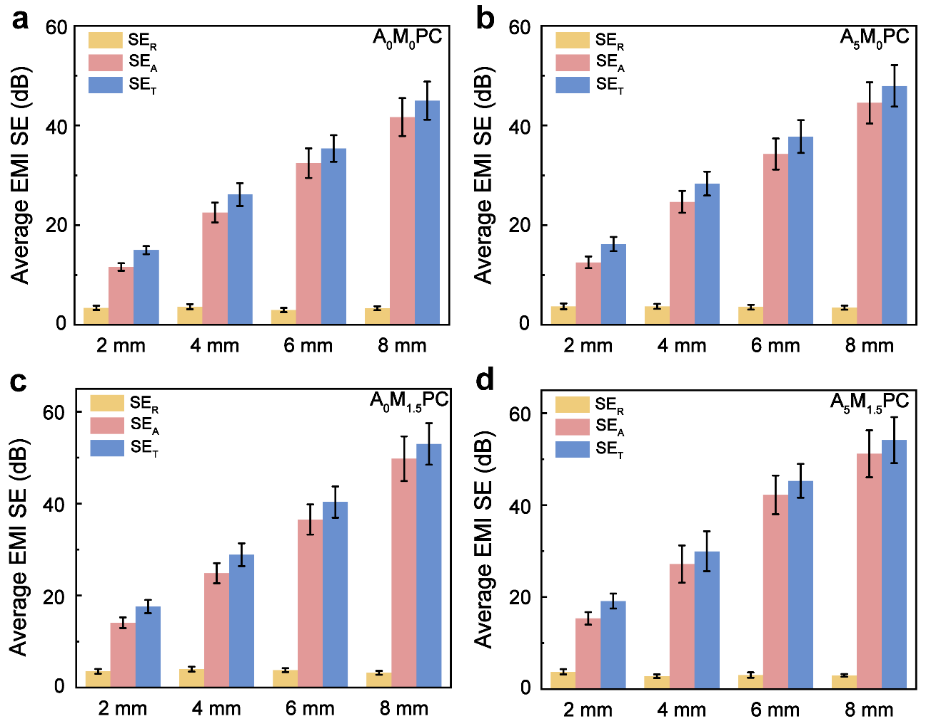


**Fig. S23** Average EMI SE_R_, SE_A_ and SE_T_ of **a** A_0_M_0_PC, **b** A_5_M_0_PC, **c** A_0_M_1.5_PC and **d** A_5_M_1.5_PC hydrogels with different thicknesses in the X-band range


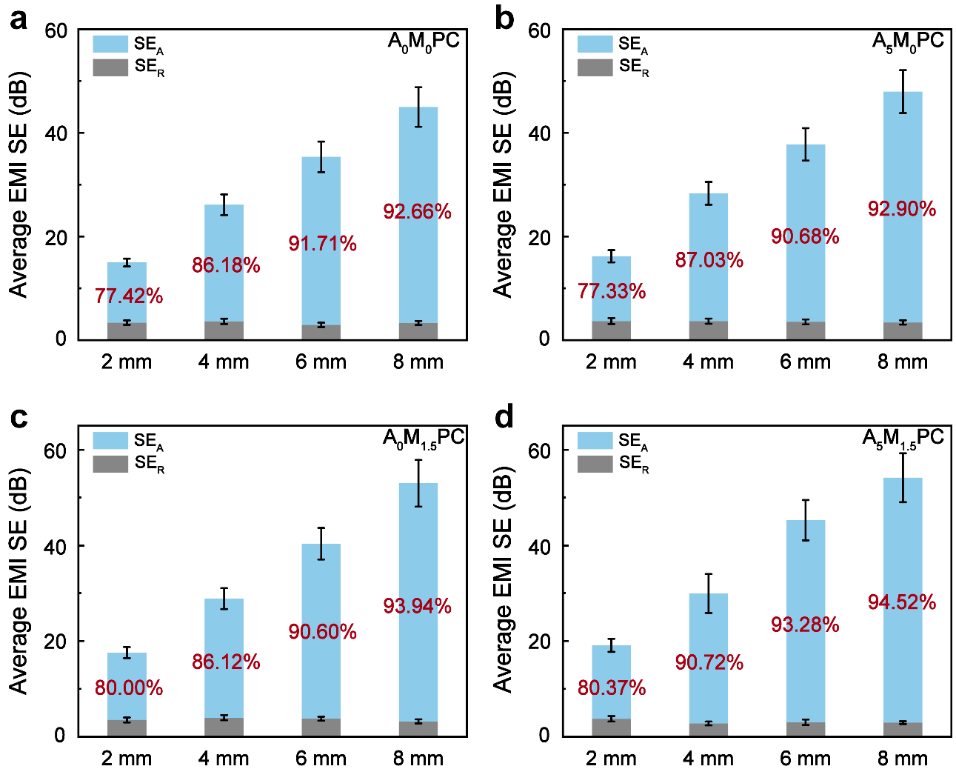


**Fig. S24** Average EMI SE_R_ and SE_A_ as well as the percentage of SE_A_ relative to SE_T_ (marked as red numbers) of **a** A_0_M_0_PC, **b** A_5_M_0_PC, **c** A_0_M_1.5_PC and **d** A_5_M_1.5_PC hydrogels with different thicknesses in the X-band range


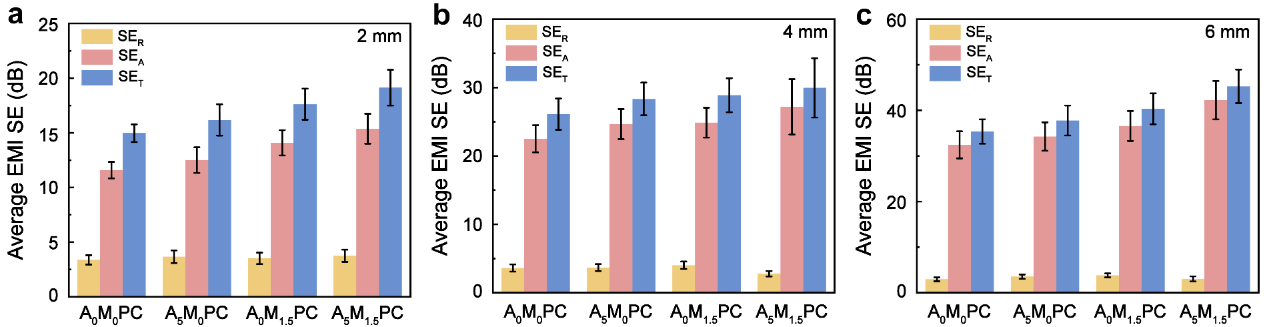


**Fig. S25** Average EMI SE_R_, SE_A_ and SE_T_ of A_x_M_y_PC hydrogels with **a** 2 mm, **b** 4 mm and **c** 6 mm in the X-band range


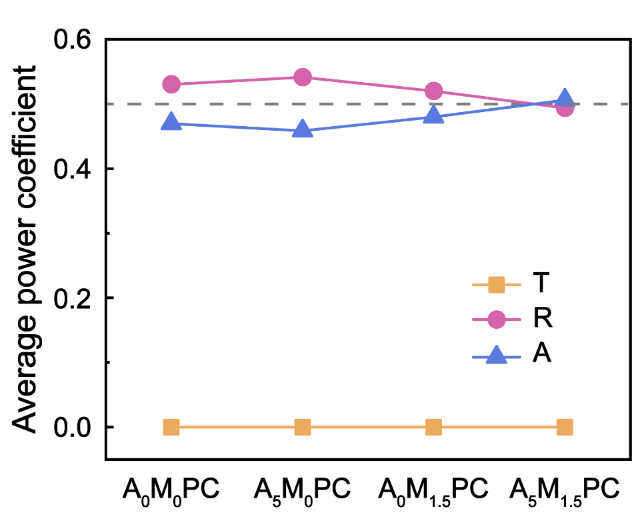


**Fig. S26** Average power coefficient of A_x_M_y_PC hydrogels in the X-band range


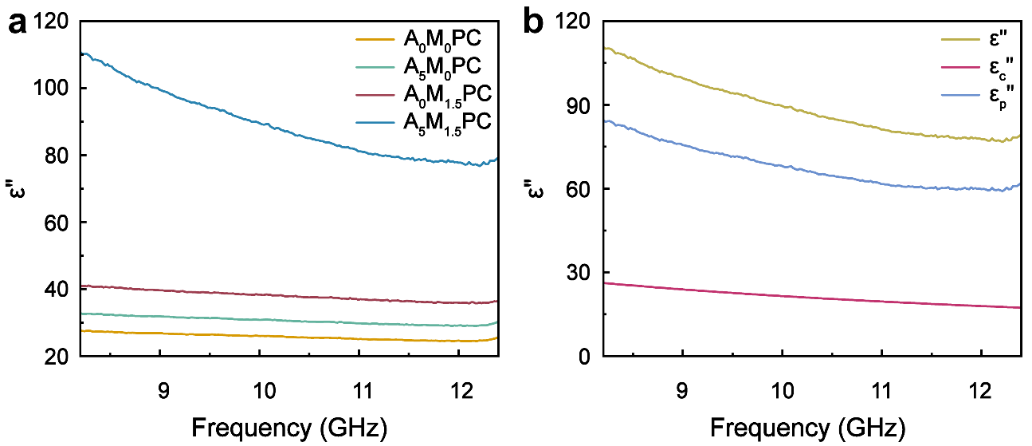


**Fig. S27** **a** ε" of A_x_M_y_PC hydrogels and **b** ε_c_" and ε_p_" of A_5_M_1.5_PC hydrogel in the X-band range

The classical Debye theoretical model was employed to calculate polarization loss and conductive loss values, thereby quantifying the impact of electromagnetic wave attenuation mechanisms. Given the non-magnetic nature of the hydrogel materials in this study, the initial analysis focused on filler effects on the imaginary dielectric constant (ε") (Fig. S27a). The polyelectrolyte hydrogels containing solely insulative ANF fillers (A_5_M_0_PC) exhibited higher ε" values compared to the filler-free counterparts (A_0_M_0_PC), yet remained lower than those incorporating exclusively MXene fillers (A_0_M_1.5_PC). Notably, the hydrogels with combined ANF/MXene fillers (A_5_M_1.5_PC) demonstrated a more pronounced ε" enhancement than the formulations containing individual filler components. The underlying mechanism stemed from distinct loss pathways: Insulative ANF primarily enhanced polarization loss through interfacial polarization and ionic path modulation, while highly conductive MXene surpassed ANF’s performance via conductive loss coupled with interfacial polarization. The synergistic incorporation of both fillers introduced multi-scale interfacial coupling and facilitated localized optimization of conductive networks, thereby achieving cooperative enhancement in electromagnetic dissipation capabilities.

The subsequent analysis based on the Debye model elucidated the loss mechanisms within the A_5_M_1.5_PC hydrogel (Fig. S27b). Polarization loss predominated over conductive loss, attributed to twofold origins. First, ANF acted as insulating spacers impeding direct contact between MXene nanosheets, suppressing conductive percolation network formation—thereby reducing overall conductivity and ultimately diminishing conductive loss. Furthermore, the coexistence of ANF and MXene introduced multi-interfacial zones that amplified heterogeneous interfacial polarization effects. These findings underscore the critical role of interfacial engineering in multiphase composite hydrogels for regulating high-frequency dielectric loss.

**Table S1** Comparison of the ratio of EMI SE_A_ to SE_T_ between this work and other references

| SE_R_/(dB) | SE_A_/(dB) | SE_T_/(dB) | SE_A_/SE_T_ | Sample | References |
| --- | --- | --- | --- | --- | --- |
| 3.3 | 62.5 | 65.8 | 94.98% | PVA/EGaInSn-8Ni | [S5] |
| 0.23 | 24.03 | 24.26 | 99.05% | M_0.9_F_3_CP-Gly/P | [S6] |
| 8.8 | 25.0 | 33.8 | 73.96% | PGMCCa | [S7] |
| 0.8 | 62.8 | 63.6 | 98.74% | POG | [S8] |
| 5.9 | 30.8 | 36.7 | 83.92% | CG6 | [S9] |
| 3.2 | 38.1 | 41.3 | 92.25% | PAM/PVA_6_/LiCl_3_ | [S10] |
| 9 | 57 | 66 | 86.36% | AgNWs-MS-based hydrogels (∼20 wt % PVA) | [S11] |
| 8.2 | 21.8 | 30 | 72.67% | C/M-10 | [S12] |
| 4.3 | 34.4 | 38.7 | 88.89% | PMP3-SSD | [S13] |
| 4.9 | 25.4 | 30.3 | 83.83% | PVA/PAA-PEDOT:PSS-TA | [S14] |
| 7.726 | 41.774 | 49.5 | 84.39% | PAAm-PHEMAA/CMC-Fe^3+^-MXene | [S15] |
| 2.97 | 51.17 | 54.14 | 94.51% | A_5_M_1.5_PC | This work |


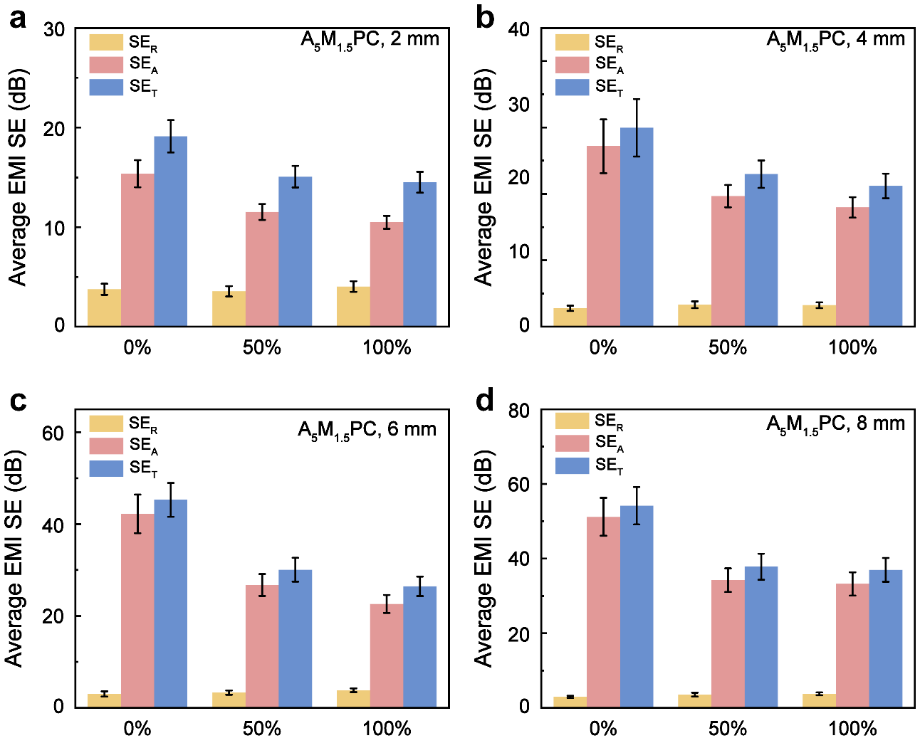


**Fig. S28** Average EMI SE_R_, SE_A_ and SE_T_ of A_5_M_1.5_PC hydrogel with **a** 2 mm, **b** 4 mm, **c** 6 mm and **d** 8 mm thickness after elongation in the X-band range


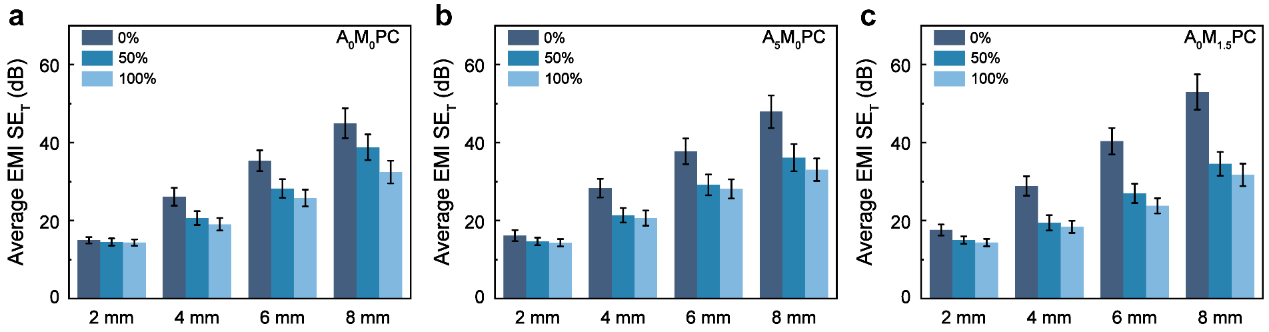


**Fig. S29** Average EMI SE_T_ of **a** A_0_M_0_PC, **b** A_5_M_0_PC and **c** A_0_M_1.5_PC hydrogels with different thicknesses after elongation in the X-band range


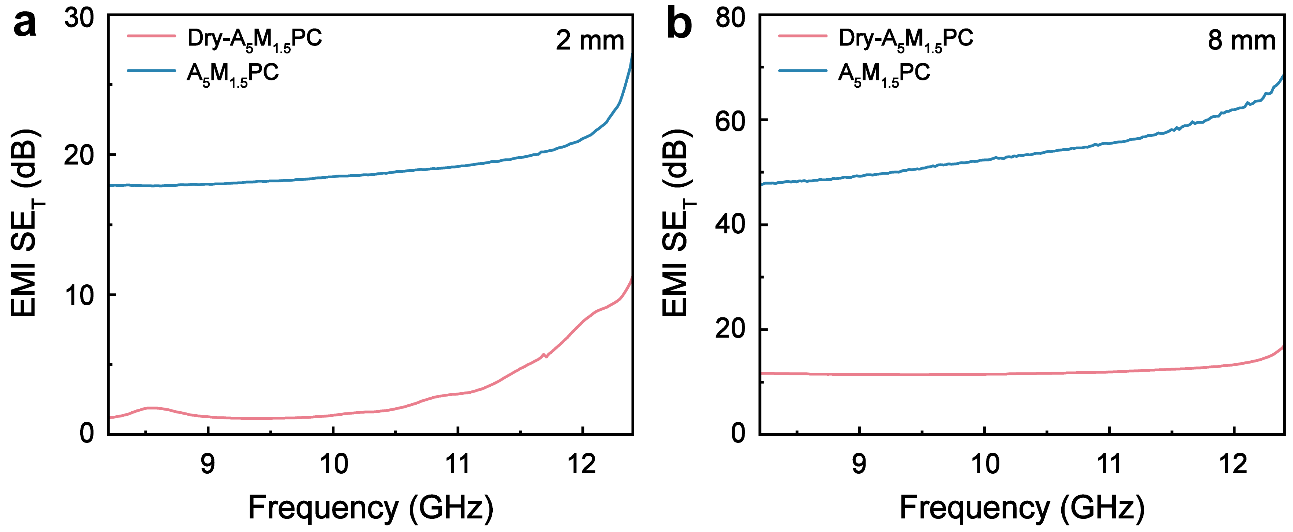


**Fig. S30** Comparison of EMI SE_T_ curves of Dry-A_5_M_1.5_PC and A_5_M_1.5_PC hydrogel with **a** 2 mm and **b** 8 mm thickness in the X-band range


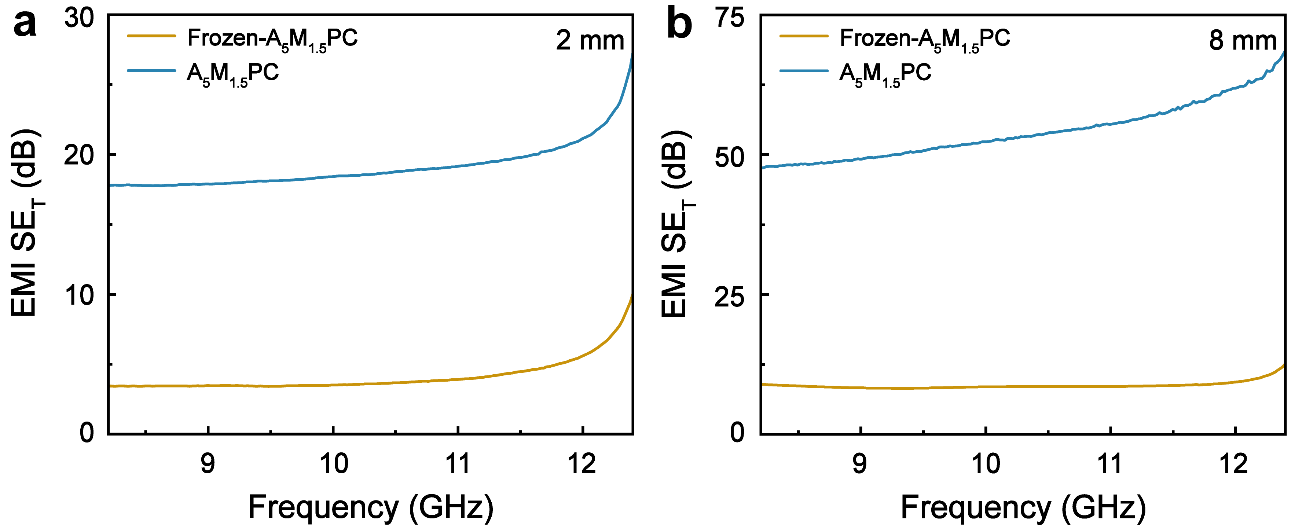


**Fig. S31** Comparison of EMI SE_T_ curves of Frozen-A_5_M_1.5_PC and A_5_M_1.5_PC hydrogel with **a** 2 mm and **b** 8 mm thickness in the X-band range


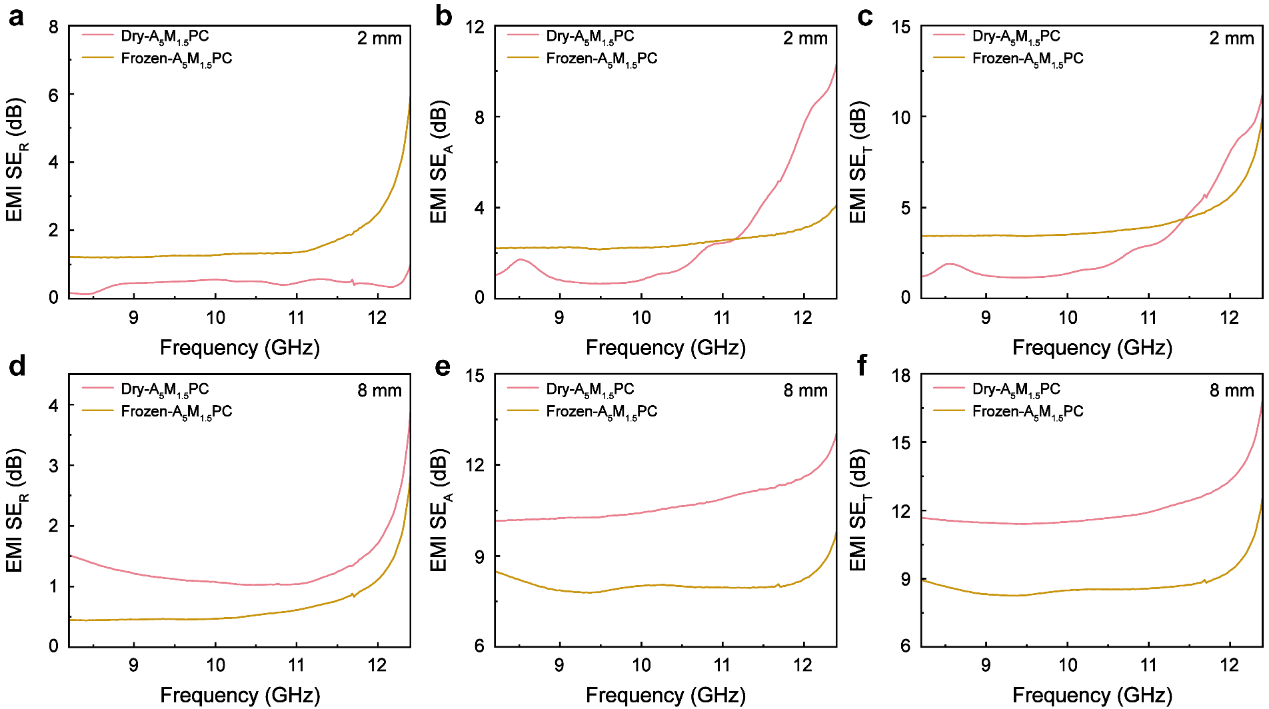


**Fig. S32** Comparison of EMI SE between Dry-A_5_M_1.5_PC and Frozen-A_5_M_1.5_PC in the thickness of 2 mm and 8 mm: **a** EMI SE_R_, 2 mm, **b** EMI SE_A_, 2 mm, **c** EMI SE_T_, 2 mm, **d** EMI SE_R_, 8 mm, **e** EMI SE_A_, 8 mm, **f** EMI SE_T_, 8 mm


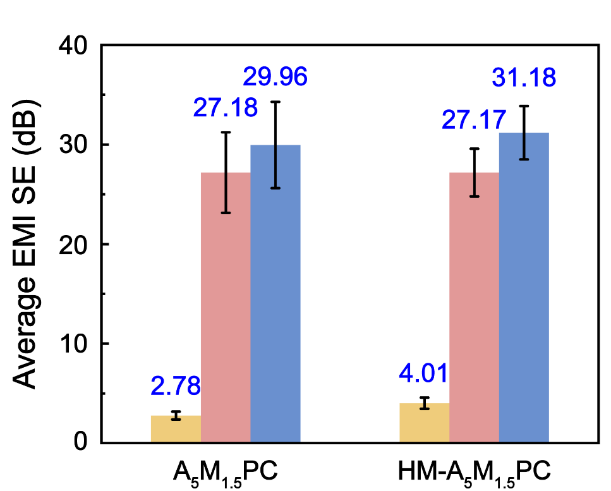


**Fig. S33** Average EMI SE of A_5_M_1.5_PC and HM-A_5_M_1.5_PC in the X-band range

The hydrogel was placed in a sealed environment with a humidifier for 3 h to test the changes in EMI SE. The hydrogel absorbed moisture, leading to increased EMI SE_T_ and SE_R_.


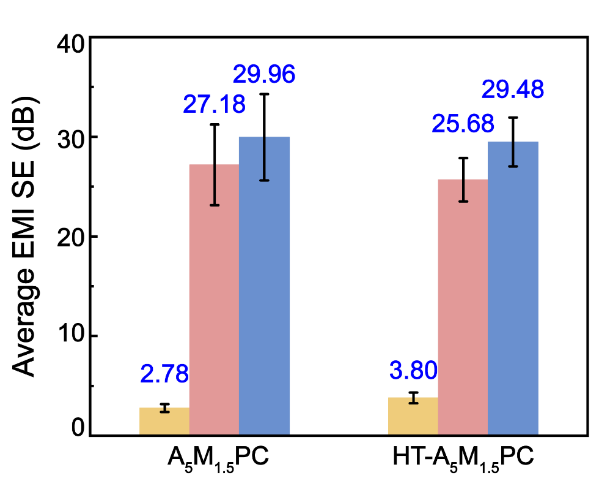


**Fig. S34** Average EMI SE of A_5_M_1.5_PC and HT-A_5_M_1.5_PC in the X-band range

The hydrogel was treated in an oven at 40 °C for 4 h, resulting in partial moisture loss, and decreased EMI SE_T_ and SE_A_. The increased EMI SE_R_ was attributed to the reduced presence of polarizable small water molecules.


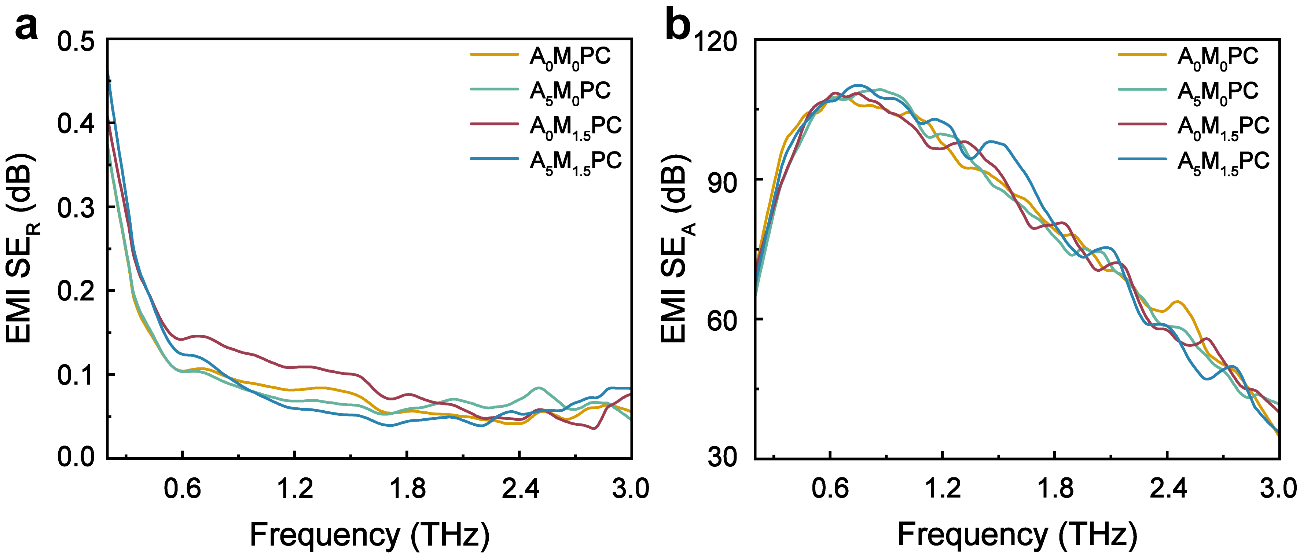


**Fig. S35** EMI **a** SE_R_ and **b** SE_A_ curves of A_0_M_0_PC, A_5_M_0_PC, A_0_M_1.5_PC and A_5_M_1.5_PC hydrogels in the THz-band range


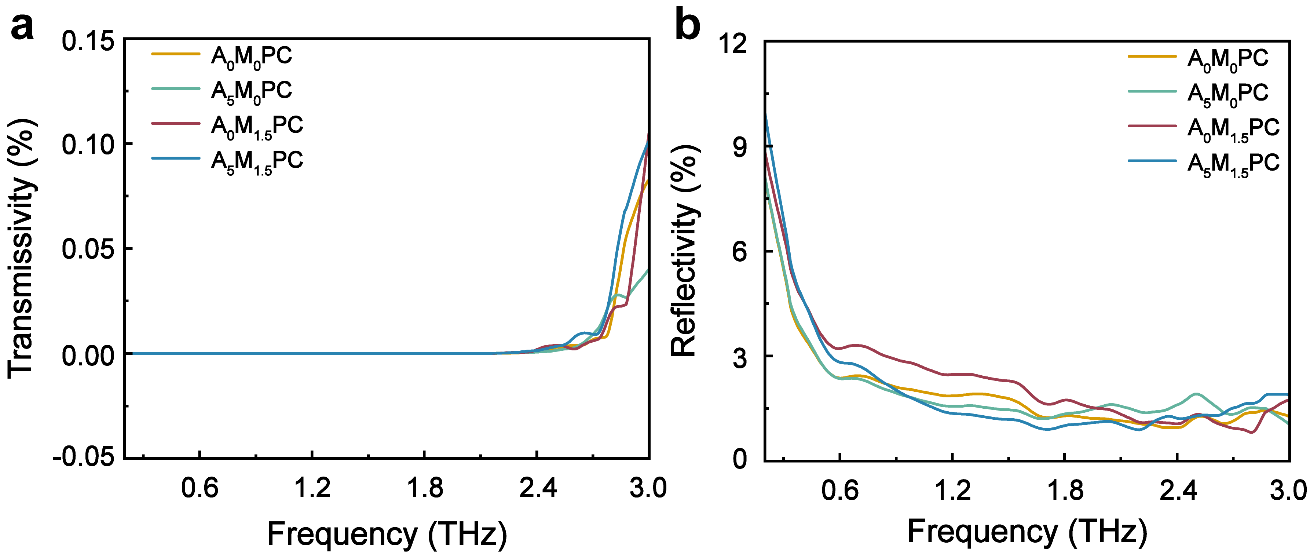


**Fig. S36** **a** Transmissivity and **b** reflectivity of A_0_M_0_PC, A_5_M_0_PC, A_0_M_1.5_PC and A_5_M_1.5_PC hydrogels in the THz-band range


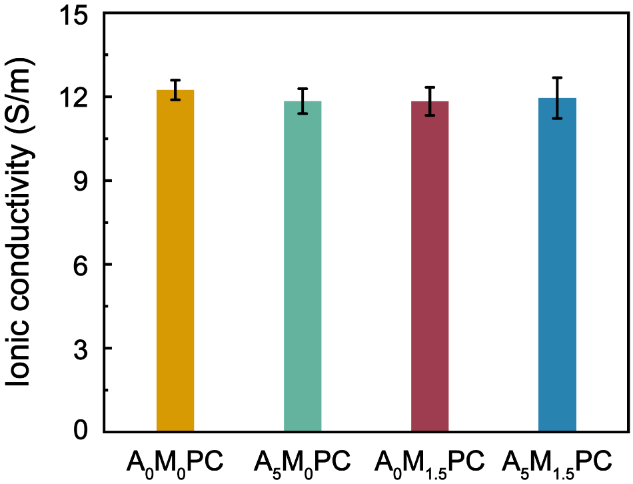


**Fig. S37** Ionic conductivity of A_0_M_0_PC, A_5_M_0_PC, A_0_M_1.5_PC and A_5_M_1.5_PC hydrogels


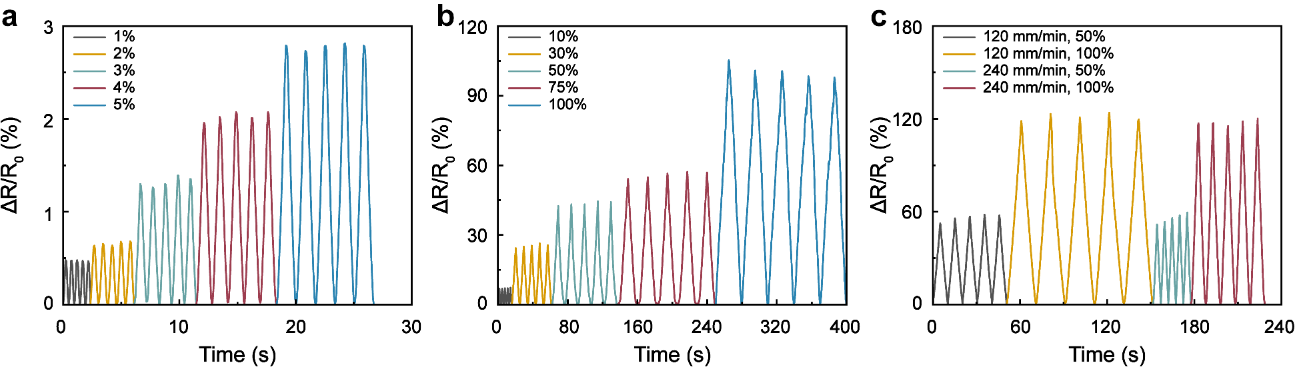


**Fig. S38** The changes in relative resistance of A_5_M_1.5_PC hydrogels in the strain range of **a** 1%~5% and **b** 10%~100% as well as **c** different stretching velocities


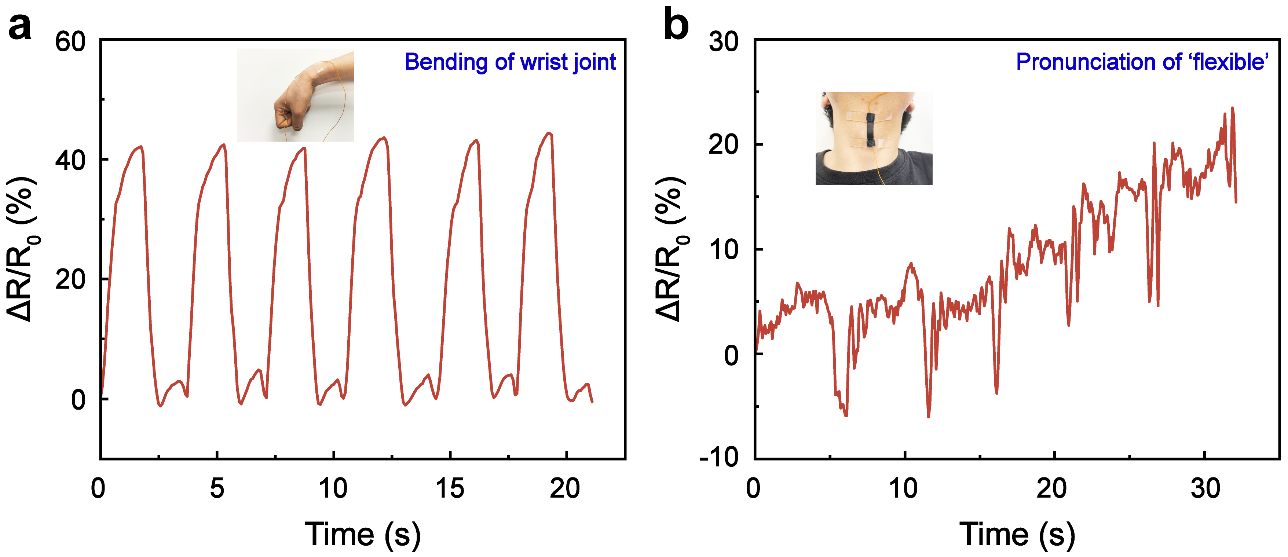


**Fig. S39** Body motion monitering of **a** bending of wrist joint and **b** pronunciation of ‘flexible’

**Supplementary References**

1. Sarycheva, Y. Gogotsi, Raman spectroscopy analysis of the structure and surface chemistry of Ti_3_C_2_T*_x_* MXene. MXenes. Jenny Stanford Publishing, (2023)., pp 333–355 <https://doi.org/10.1201/9781003306511-16>
2. J. Isailović, A. Oberlintner, U. Novak, M. Finšgar, F.M. Oliveira et al., Study of chitosan-stabilized Ti_3_C_2_T *_x_* MXene for ultrasensitive and interference-free detection of gaseous H_2_O_2_. ACS Appl. Mater. Interfaces **15**(26), 31643–31651 (2023). <https://doi.org/10.1021/acsami.3c05314>
3. Yang, L. Wang, M. Zhang, J. Luo, X. Ding, Timesaving, high-efficiency approaches to fabricate aramid nanofibers. ACS Nano **13**(7), 7886–7897 (2019). <https://doi.org/10.1021/acsnano.9b02258>
4. J. Wang, Y. Lin, A. Mohamed, Q. Ji, H. Jia, High strength and flexible aramid nanofiber conductive hydrogels for wearable strain sensors. J. Mater. Chem. C **9**(2), 575–583 (2021). <https://doi.org/10.1039/D0TC02983A>
5. Zhao, Z. Bai, H. Lv, Z. Yan, Y. Du et al., Self-healing liquid metal magnetic hydrogels for smart feedback sensors and high-performance electromagnetic shielding. Nanomicro Lett. **15**(1), 79 (2023). <https://doi.org/10.1007/s40820-023-01043-3>
6. Y. He, J. Chen, Y. Qian, Y. Wei, C. Wang et al., Organohydrogel based on cellulose-stabilized emulsion for electromagnetic shielding, flame retardant, and strain sensing. Carbohydr. Polym. **298**, 120132 (2022). <https://doi.org/10.1016/j.carbpol.2022.120132>
7. R. Zhang, D. Xie, C. Zhang, Z. Xu, Y. Fang et al., A highly stretchable, self-adhesive, anti-freezing, and highly sensitive dual-network conductive hydrogel sensor for multifunctional electronic skin. J. Mater. Chem. A **11**(45), 24608–24617 (2023). <https://doi.org/10.1039/D3TA04980A>
8. L. Meng, S. Yang, Y. Ma, Y. Zou, Y. Zhang et al., Porous polypyrrole nanotube-based organohydrogels: versatile materials for robust electromagnetic interference shielding in harsh environments. Chem. Eng. J. **479**, 147643 (2024). <https://doi.org/10.1016/j.cej.2023.147643>
9. R. Yin, C. Zhang, J. Shao, Y. Chen, A. Yin et al., Integration of flexible, recyclable, and transient gelatin hydrogels toward multifunctional electronics. J. Mater. Sci. Technol. **145**, 83–92 (2023). <https://doi.org/10.1016/j.jmst.2022.10.047>
10. S. Yuan, T. Dai, X. Jiang, H. Zou, P. Liu, Transparent and environmentally adaptive semi-interpenetrating network hydrogels for electromagnetic interference shielding. ACS Appl. Polym. Mater. **5**(10), 8406–8414 (2023). <https://doi.org/10.1021/acsapm.3c01381>
11. Y. Yang, N. Wu, B. Li, W. Liu, F. Pan et al., Biomimetic porous MXene sediment-based hydrogel for high-performance and multifunctional electromagnetic interference shielding. ACS Nano **16**(9), 15042–15052 (2022). <https://doi.org/10.1021/acsnano.2c06164>
12. Y. Bai, S. Bi, W. Wang, N. Ding, Y. Lu et al., Biocompatible, stretchable, and compressible cellulose/MXene hydrogel for strain sensor and electromagnetic interference shielding. Soft Mater. **20**(4), 444–454 (2022). <https://doi.org/10.1080/1539445x.2022.2081580>
13. T.-Y. Zhu, W.-J. Jiang, S. Wu, Z.-J. Huang, Y.-L. Liu et al., Multifunctional MXene/PEDOT: PSS-based phase change organohydrogels for electromagnetic interference shielding and medium-low temperature infrared stealth. ACS Appl. Mater. Interfaces **16**(12), 15372–15382 (2024). <https://doi.org/10.1021/acsami.4c01001>
14. Z. Zhou, W. Yuan, X. Xie, A stretchable and adhesive composite hydrogel containing PEDOT: PSS for wide-range and precise motion sensing and electromagnetic interference shielding and as a triboelectric nanogenerator. Mater. Chem. Front. **6**(22), 3359–3368 (2022). <https://doi.org/10.1039/D2QM00690A>
15. K. Fan, K. Li, L. Han, Z. Yang, J. Yang et al., Multifunctional double-network Ti_3_C_2_T*_x_* MXene composite hydrogels for strain sensors with effective electromagnetic interference and UV shielding properties. Polymer **273**, 125865 (2023). <https://doi.org/10.1016/j.polymer.2023.125865>
